# The Gut Microbiota as a Mediator in the Relationship Between Dietary Patterns and Depression

**DOI:** 10.1002/mco2.70562

**Published:** 2026-01-20

**Authors:** Adrián Hernández‐Cacho, Jiaqi Ni, Jesús F. García‐Gavilán, Prokopis Konstanti, Clara Belzer, Jesús Vioque, Dolores Corella, Montserrat Fitó, Josep Vidal, Laura Torres‐Collado, Oscar Coltell, Nancy Babio, Javier Hernando‐Redondo, Isabel Moreno‐Indias, Miguel Ruiz‐Canela, Francisco J. Tinahones, Jordi Salas‐Salvadó

**Affiliations:** ^1^ Universitat Rovira i Virgili Departament De Bioquímica i Biotecnologia Alimentació, Nutrició, Desenvolupament i Salut Mental ANUT‐DSM Reus Spain; ^2^ Institut D'investigació Sanitària Pere Virgili (IISPV) Reus Spain; ^3^ CIBER de Fisiopatología de la Obesidad y Nutrición Instituto de Salud Carlos III Madrid Spain; ^4^ Laboratory of Microbiology Wageningen University Wageningen the Netherlands; ^5^ CIBER De Epidemiología y Salud Pública (CIBERESP) Instituto De Salud Carlos III Madrid Spain; ^6^ Instituto De Investigación Sanitaria y Biomédica de Alicante Universidad Miguel Hernández (ISABIAL‐UMH) Alicante Spain; ^7^ Department of Preventive Medicine University of Valencia Valencia Spain; ^8^ Unit of Cardiovascular Risk and Nutrition Institut Hospital del Mar de Investigaciones Médicas Municipal D‘Investigació Médica (IMIM) Barcelona Spain; ^9^ CIBER Diabetes y Enfermedades Metabólicas (CIBERDEM) Instituto De Salud Carlos III (ISCIII) Madrid Spain; ^10^ Department of Endocrinology Institut D‘Investigacions Biomédiques August Pi Sunyer (IDIBAPS) Hospital Clinic University of Barcelona Barcelona Spain; ^11^ Department of Computer Languages and Systems Jaume I University Castellón Spain; ^12^ Department of Endocrinology and Nutrition Instituto De Investigación Biomédica de Málaga – Plataforma Bionand IBIMA Hospital Universitario Virgen De La Victoria Málaga Spain; ^13^ Department of Preventive Medicine and Public Health University of Navarra Pamplona Spain; ^14^ IdiSNA, Navarra Institute for Health Research Pamplona Spain

**Keywords:** gut microbiota, depression, dietary patterns, Mediterranean diet, microbiome mediation, mental health, 16S rRNA sequencing, gut–brain axis

## Abstract

The interplay between diet, gut microbiota, and depressive symptoms is increasingly recognized, but underlying mechanisms remain unclear. We investigated whether adherence to several dietary patterns relates to gut microbial signatures and whether these profiles are associated with depressive symptoms in an elderly Mediterranean cohort. In 644 participants, 16S ribosomal RNA gene sequencing and dietary intake from a food‐frequency questionnaire were obtained at baseline and 1‐year follow‐up. Adherence scores were computed for the Mediterranean diet adherence score (MEDAS), energy‐reduced MEDAS (erMEDAS), Dietary Approaches to Stop Hypertension (DASH), Healthy Plant‐Based Diet Index (HPDI), Unhealthy Plant‐Based Diet Index (UPDI), and Western Diet Score (WESTDIET). Healthy patterns (erMEDAS, MEDAS, DASH, HPDI) were associated with 22, 28, 24, and 16 genera, of which 82%, 75%, 79%, and 88% showed a protective profile (more abundant with lower, or less abundant with higher, depressive symptoms). UPDI and WESTDIET were associated with 20 and 27 genera, but only 25% and 26% were protective. Mediation analyses indicated that gut microbiota mediated the associations of MEDAS (ACME = –0.066, *p* = 0.006) and erMEDAS (ACME = –0.029, *p* = 0.011) with depressive symptoms. This study is among the first to test whether diet shapes a microbiota signature that mediates the diet–depression relationship, adding mechanistic insight into diet–mental health research.

## Graphical abstract

**Healthy dietary patterns are associated with gut microbial genera linked to lower depressive symptoms**. In 644 older adults from the PREDIMED‐Plus cohort, 16S rRNA sequencing and dietary scoring revealed that taxa associated with a better diet quality were also more abundant in individuals with fewer depressive symptoms. Mediation analysis showed that gut microbiota significantly mediates the link between Mediterranean diet adherence and depression scores. These findings highlight a potential microbiome‐driven pathway connecting diet and mental health.

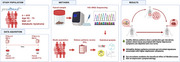

## 1 Introduction

Diet is a key modulator of both gut microbiota and mental health, serving as a bridge between physical and psychological well‐being [1]. What humans eat not only determines the diversity and functionality of the microorganisms inhabiting their gut but also impacts brain health through complex and interconnected pathways [2, 3]. The gut microbiota produces metabolites that influence inflammation, neurotransmitter synthesis, and gut–brain signaling, key processes implicated in mood regulation and cognitive function [4, 5]. Simultaneously, dietary habits directly affect mental health by altering nutrient availability, immune responses, and hormonal balance [6]. This intricate interplay highlights the potential to transform how we approach the prevention and management of mental health disorders by linking nutrition, gut microbial health, and mental well‐being in a dynamic and promising field of study.

Observational and interventional evidence indicate that overall diet quality relates to depressive symptoms and risk of depression [7]. Higher adherence to Mediterranean‐style diets has been associated with lower odds of depression across cohorts and systematic reviews, and several randomized trials have reported improvements in depressive symptoms with dietary support [8, 9]. In contrast, greater adherence to Western‐type or ultraprocessed‐food‐rich patterns has been linked to higher depressive symptom burden and incident depression in multiple cohorts and recent reviews [10, 11].

Dietary patterns have been related to anxiety and depression and have been shown to impact the diversity and function of gut microbiota, with certain “healthy” diets decreasing depressive symptomatology or risk of depression and promoting beneficial microbial communities that produce metabolites implicated in anti‐inflammatory and neuroprotective processes [12]. Conversely, “unhealthy” diets high in processed foods and low in fiber are associated higher risk of depression and with microbial dysbiosis, inflammation, and disruptions to the gut–brain axis [13, 14]. These findings underscore the importance of examining diet as a whole rather than focusing on isolated nutrients, as dietary patterns provide a more comprehensive framework for understanding their influence on gut microbiota and downstream effects on health.

Although growing evidence supports the relationships between dietary patterns, gut microbiota, and depression, the mechanisms underlying these interactions remain unclear [15], and few studies have explored the mechanisms involving gut microbiota in the diet–depression relationship [16]. Addressing these gaps requires integrating data on dietary patterns, microbial composition, and mental health outcomes to clarify how these factors interact and influence each other.

This study aims to investigate the association between adherence to dietary patterns, gut microbiota diversity and profile, and depressive symptomatology, with a particular focus on the mediating role of the gut microbiota in the diet–depression relationship. By analyzing these interactions, this research seeks to elucidate potential biological pathways connecting diet, microbiota, and mental health. These findings could inform the development of personalized dietary strategies targeting gut and brain health to reduce the burden of depression and improve overall well‐being.

## 2 Results

### 2.1 Baseline Characteristics of the Study Population

Table 1 presents the baseline characteristics of the 644 participants included in this study.

**TABLE 1:** Baseline characteristics of the PREDIMED‐Plus participants included in this substudy.

	*N* = 644^a^
BDI‐II	7 (3, 12)
Sociodemographic variables	
Female	304 (47%)
Age	65 (61, 68)
Civil status	
Married	496 (77%)
Other	148 (23%)
Education	
Primary school or less	342 (53%)
Secondary school	182 (28%)
Higher education	120 (19%)
Employment	
Active	135 (21%)
Unemployed	121 (19%)
Retired	388 (60%)
Lifestyle factors and medication use	
Alcohol consumption (g/day)	5 (1, 15)
Smoking status	
Current smoker	83 (13%)
Ex‐smoker	250 (39%)
Never smoker	311 (48%)
Antidepressant treatment	68 (11%)
Anthropometry	
BMI category	
Overweight	159 (25%)
Obese	485 (75%)
Metabolic syndrome components	
Elevated waist circumference	618 (96%)
Elevated triglycerides/lipid‐lowering treatment	342 (53%)
Reduced HDL‐C/treatment	249 (39%)
Elevated blood pressure/antihypertensive treatment	605 (94%)
Elevated fasting glucose/antidiabetic treatment	482 (75%)
Number of components met	
Exactly 3	432 (67%)
Exactly 4	136 (21%)
All 5	76 (12%)
Dietary patterns score	
erMEDAS	8 (6, 10)
MEDAS	8 (7, 10)
DASH	24 (20, 27)
HPDI	54 (49, 60)
WESTDIET	36 (31, 40)
UPDI	55 (50, 60)

Abbreviations: BDI‐II, Beck Depression Inventory‐II; BMI, body mass index; DASH, Dietary Approaches to Stop Hypertension; erMEDAS, Energy‐Reduced Mediterranean Diet Adherence Screener; HPDI, Healthful Plant‐Based Diet Index; MEDAS, Mediterranean Diet Adherence Screener; UPDI, Unhealthful Plant‐Based Diet Index; WESTDIET, Western Diet Index.

^a^ *n* (%); median (IQR).

The median Beck Depression Inventory‐II (BDI‐II) score was 7 (IQR: 3–12). The sample included 47% women, with a median age of 65 years (IQR: 61–68). Median alcohol consumption was 5 g/day (IQR: 1–15 g/day), and 13% were current smokers. Antidepressant medication use was reported by 11% of participants. Median scores for dietary patterns were erMEDAS: 8 (IQR: 6–10), MEDAS: 8 (IQR: 7–10), DASH: 24 (IQR: 20–27), HPDI: 54 (IQR: 49–60), WESTDIET: 36 (IQR: 31–40), and UPDI: 55 (IQR: 50–60).

Baseline characteristics of the population included in the present study, the total population of the four recruiting centers included in this study, and all the PREDIMED‐Plus study population are described in Table S1. The variables are well balanced among the three populations. Baseline values and 1‐year changes for dietary pattern scores, the microbiota score of each pattern, and BDI‐II are shown in Table S2.

### 2.2 Association Between Adherence to Different Dietary Patterns and Depression Symptomatology

Table 2 presents the associations between dietary pattern scores and BDI‐II, as well as between changes in dietary pattern scores and changes in BDI‐II over time. Two sets of models were conducted: Linear mixed effect models evaluating longitudinal associations between dietary pattern scores and BDI‐II, and change‐based associations between changes in dietary pattern scores and changes in BDI‐II.

**TABLE 2:** Longitudinal associations between dietary pattern scores and BDI, and between their changes after 1 year.

Dietary pattern score against BDI‐II (1)			ΔDietary pattern score against ΔBDI‐II (2)		
	*β* (95%CI)	*p*‐value		*β* (95%CI)	*p*‐value
Energy‐Reduced Mediterranean Diet Adherence Score (erMEDAS)					
Model 1	**−0.291 (−0.376, −0.206)**	<0.01	Model 1	**−0.183 (−0.350, −0.015)**	<0.01
Model 2	**−0.156 (−0.271, −0.042)**	<0.01	Model 2	**−0.225 (−0.356, −0.094)**	<0.01
Model 3	**−0.166 (−0.284, −0.047)**	<0.01	Model 3	**−0.168 (−0.304, −0.031)**	0.02
Mediterranean Diet Adherence Score (MEDAS)					
Model 1	−**0.438 (**−**0.569,** −**0.308)**	**<0.01**	Model 1	−**0.188 (**−**0.468,** −**0.091)**	**0.02**
Model 2	−**0.208 (**−**0.388,** −**0.028)**	**0.02**	Model 2	−**0.254 (**−**0.473,** −**0.036)**	**0.02**
Model 3	−**0.203 (**−**0.386,** −**0.019)**	**0.03**	Model 3	−0.170 (−0.395, 0.055)	0.14
Dietary Approaches to Stop Hypertension (DASH)					
Model 1	−**0.073 (**−**0.138,** −**0.008)**	**0.03**	Model 1	−**0.109 (**−**0.211,** −**0.006)**	**<0.01**
Model 2	−**0.100 (**−**0.165,** −**0.035)**	**<0.01**	Model 2	−**0.140 (**−**0.220,** −**0.059)**	**<0.01**
Model 3	−**0.106 (**−**0.172,** −**0.039)**	**<0.01**	Model 3	−**0.123 (**−**0.207,** −**0.039)**	**<0.01**
Healthy Plant‐Based Diet Index (HPDI)					
Model 1	−**0.061 (**−**0.103,** −**0.020)**	**<0.01**	Model 1	−**0.071 (**−**0.134,** −**0.007)**	**<0.01**
Model 2	−**0.054 (**−**0.095,** −**0.013)**	**0.01**	Model 2	−**0.070 (**−**0.120,** −**0.020)**	**<0.01**
Model 3	−**0.055 (**−**0.097,** −**0.014)**	**<0.01**	Model 3	−**0.051 (**−**0.103, 0.000)**	**0.05**
Western Diet Score (WESTDIET)					
Model 1	**0.083 (0.039, 0.128)**	**<0.01**	Model 1	**0.085 (0.018, 0.152)**	**<0.01**
Model 2	**0.083 (0.039, 0.127)**	**<0.01**	Model 2	**0.105 (0.052, 0.157)**	**<0.01**
Model 3	**0.082 (0.037, 0.127)**	**<0.01**	Model 3	**0.085 (0.030, 0.140)**	**<0.01**
Unhealthy Plant‐Based Diet Index (UPDI)					
Model 1	**0.067 (0.018, 0.117)**	**<0.01**	Model 1	**0.060 (**−**0.019, 0.139)**	**0.02**
Model 2	**0.055 (0.005, 0.105)**	**0.03**	Model 2	**0.075 (0.012, 0.138)**	**0.02**
Model 3	0.049 (−0.002, 0.099)	0.06	Model 3	0.063 (−0.002, 0.129)	0.06

*Note*: The table presents two sets of models: (1) dietary pattern scores against BDI‐II and (2) changes in dietary pattern scores against changes in BDI‐II. For the dietary pattern against BDI‐II models, Model 1 included only a random intercept for participants. Model 2 adjusted for visit, gender, age, and study center. Model 3 further adjusted for BMI, employment status, marital status, education level, alcohol consumption (g/day), hypertension prevalence, diabetes prevalence, intervention group, smoking status, and antidepressant use. For the models assessing changes in dietary pattern scores against changes in BDI‐II, Model 1 included the change in dietary pattern score as exposure, adjusted for its baseline level and baseline BDI‐II. Model 2 additionally adjusted for sex and age. Model 3 further adjusted for intervention group, study center, BMI, employment status, marital status, education level, changes in alcohol consumption (g/day), hypertension prevalence, diabetes prevalence, smoking status, and antidepressant use.

Abbreviations: BDI‐II, Beck Depression Inventory‐II; CI, confidence interval.

In our longitudinal models, higher adherence to erMEDAS (*β*: −0.17; 95% CI: −0.28 to −0.05; *p* < 0.01), MEDAS (*β*: −0.20; 95% CI: −0.39 to −0.02; *p* = 0.03), DASH (*β*: −0.11; 95% CI: −0.17 to −0.04; *p* < 0.01) and HPDI (*β*: −0.06; 95% CI: −0.10 to −0.01; *p* < 0.01) was associated with lower BDI‐II scores, whereas greater adherence to WESTDIET (*β*: 0.08; 95% CI: 0.04 to 0.13; *p* < 0.01) and UPDI (β: 0.05; 95% CI: −0.002 to 0.10; *p* = 0.06) was associated with higher BDI‐II scores. These associations were consistent across Models 1–3, with effect sizes attenuated after adjusting for additional covariates. In the change‐score analysis, increases in erMEDAS (*β*: −0.17; 95% CI: −0.30 to −0.03; *p* = 0.02), DASH (*β*: −0.12; 95% CI: −0.21 to −0.04; *p* < 0.01), and HPDI (*β*: −0.05; 95% CI: −0.10 to 0.00; *p* = 0.05) were associated with reductions in BDI‐II over time, while adherence to WESTDIET (*β*: 0.09; 95% CI: 0.03 to 0.14; *p* < 0.01) remained positively associated with changes in BDI‐II scores. However, for changes in MEDAS (*β*: −0.17; 95% CI: −0.40 to 0.06; *p* = 0.14) and UPDI (*β*: 0.06; 95% CI: −0.002 to 0.13; *p* = 0.06), the associations with changes in BDI‐II were not consistent across all models. The effect sizes were generally attenuated after adjusting for potential confounders, particularly in Model 3. Across the six indices, only HPDI showed evidence of a diet × sex interaction; this finding did not change the direction or significance of the primary results and is noted for completeness.

### 2.3 Associations of Dietary Patterns with Gut Microbial Alpha Diversity

**FIGURE 1:**
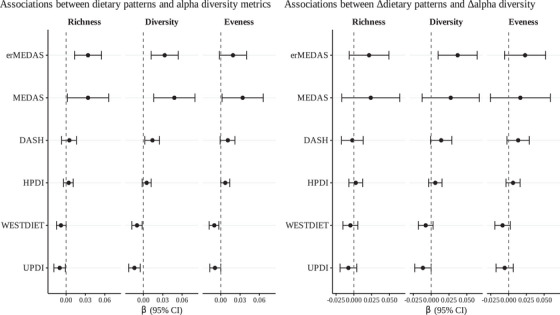
Forest plot of the longitudinal associations between dietary pattern scores and alpha diversity indices (left) and 1‐year changes in dietary pattern scores and changes in alpha diversity indices (right). Each point represents the estimated *β* coefficient for a given dietary pattern, with horizontal error bars indicating the 95% confidence interval. A dashed vertical line at *β* = 0 denotes the null association. Longitudinal values obtained from linear mixed effect models of dietary pattern scores (exposure) and alpha diversity indices (outcome) adjusted for visit, gender, age, study center, BMI, employment status, marital status, education level, alcohol consumption (g/day), hypertension prevalence, diabetes prevalence, intervention group, smoking status, antidepressant use and participant ID as random intercept. Change based values obtained from linear models of changes in dietary patterns scores (exposure) and changes in each alpha diversity index (outcome) adjusted for baseline dietary pattern score, baseline gut microbiota score, gender, age, study center, BMI, employment status, marital status, education level, changes in alcohol consumption (g/day), hypertension prevalence, diabetes prevalence, intervention group, smoking status, antidepressant use. DASH, Dietary Approaches to Stop Hypertension; erMEDAS, Energy‐Reduced Mediterranean Diet Adherence Screener; HPDI, Healthful Plant‐Based Diet Index; MEDAS, Mediterranean Diet Adherence Screener; UPDI, Unhealthful Plant‐Based Diet Index; WESTDIET, Western Diet Index.

In the association analyses between dietary pattern scores and alpha diversity indices, erMEDAS and MEDAS each showed significant positive associations with both microbial richness and diversity. MEDAS additionally exhibited a positive relationship with evenness. In contrast, UPDI was significantly negatively associated with microbiota richness and diversity, and WESTDIET was significantly negatively associated with diversity and evenness. Finally, DASH adherence was positively associated with diversity. In analyses of within‐subject changes, increases in erMEDAS were significantly positively associated with increases in microbial diversity, whereas increases in UPDI showed the opposite trend (Figure 1).

### 2.4 Gut Microbiota Genus Selected for the Gut Microbiota Dietary Pattern Scores

The penalized regression robustly selected the following number of genera targeting each dietary pattern: MEDAS was associated with 28 taxa (16 negatively, 12 positively), erMEDAS with 22 taxa (7 negatively, 15 positively), DASH with 24 taxa (10 negatively, 14 positively), HPDI with 16 taxa (8 negatively, 8 positively), WESTDIET with 27 taxa (19 negatively, 8 positively), and UPDI with 20 taxa (13 negatively, 7 positively).

Four taxa, *Eubacterium xylanophilum group, Hungatella, Oscillospiraceae NK4A214 group*, and *Lachnospiraceae NK4A136* group, were consistently associated with healthy dietary patterns (MEDAS, erMEDAS, DASH, and HPDI).

In contrast, taxa commonly associated with unhealthy dietary patterns (WESTDIET and UPDI) were all negatively associated and included *Unclassified UCG‐010, Eubacterium xylanophilum group, Herbinix, Lachnospiraceae UCG‐003, Oscillospiraceae NK4A214 group, Christensenellaceae R‐7 group, Subdoligranulum, Lachnospiraceae FCS020 group, Eubacterium ruminantium group, and Lachnospiraceae UCG‐001*.

**FIGURE 2:**
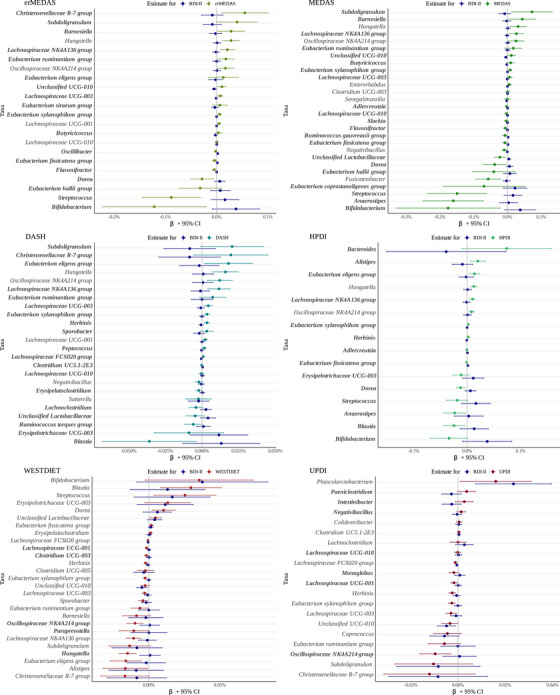
The figure illustrates the direction of associations between dietary patterns and taxa, and between BDI‐II and the same taxa. Taxa enriched in individuals with higher adherence to healthy dietary patterns (erMEDAS, MEDAS, DASH, HPDI) tended to be depleted in those with higher depressive symptomatology and vice versa, meaning that taxa enhanced by these diets were typically reduced in individuals with higher depression symptomatology, while those decreased by these diets were more abundant in individuals with higher depression symptomatology. In contrast, taxa associated with unhealthy dietary patterns (WESTDIET, UPDI) largely showed aligned associations with BDI‐II, indicating that taxa promoted by these diets were also more abundant in individuals with higher depression symptomatology, whereas those reduced by these diets were typically more abundant in individuals with lower depression symptomatology. Taxa that exhibited inverse associations with dietary patterns and BDI‐II are highlighted in bold. Values obtained from linear mixed effect models of dietary index score (exposure) and each taxon (outcome) adjusted for visit, gender, age, study center, BMI, employment status, marital status, education level, alcohol consumption (g/day), hypertension prevalence, diabetes prevalence, intervention group, smoking status, antidepressant use, and participant ID as random intercept. BDI‐II, Beck Depression Inventory‐II; CI, confidence interval; DASH, Dietary Approaches to Stop Hypertension; erMEDAS, Energy‐Reduced Mediterranean Diet Adherence Screener; HPDI, Healthful Plant‐Based Diet Index; MEDAS, Mediterranean Diet Adherence Screener; UPDI, Unhealthful Plant‐Based Diet Index; UCG, Unclassified *Clostridia* Genus; WESTDIET, Western Diet Index.

Additionally*, Eubacterium xylanophilum group and Oscillospiraceae NK4A214 group* were identified in both healthy and unhealthy dietary patterns, but with opposing associations, being positively associated with healthy dietary patterns, while negatively associated with unhealthy dietary patterns. The bacterial genus that better predicts adherence to each dietary pattern according to the LMM‐LASSO can be seen in Figure 2 and Tables S3–S8.

### 2.5 Gut Microbiota Signatures of Dietary Patterns Align or Oppose Those Associated with Depression

When analyzing the associations between dietary patterns, gut microbiota, and BDI‐II scores, a distinct inverse pattern emerged for healthy dietary patterns (erMEDAS, MEDAS, DASH, HPDI). Taxa that were more abundant in individuals adhering to these diets were typically less abundant in those with higher depressive symptomatology, and conversely, taxa that were reduced in individuals with high adherence to healthy diets were often enriched in those with higher BDI‐II scores (Figure 2). Specifically, this pattern was observed in 18 out of 22 taxa for erMEDAS, 21 out of 28 for MEDAS, 19 out of 24 for DASH, and 14 out of 16 for HPDI.

In contrast, for unhealthy dietary patterns (WESTDIET, UPDI), most taxa showed the same trend in their associations with both diet and BDI‐II. This pattern was observed in only 5 out of 27 taxa for WESTDIET and 7 out of 20 for UPDI, meaning that most of the taxa associated with these dietary patterns changed in the same direction as in individuals with higher BDI‐II scores (Figure 2). Taxa that were more abundant in individuals adhering to unhealthy diets were also more abundant in those with higher BDI‐II scores, while those that were less abundant in individuals following these diets were also less abundant in individuals with higher BDI‐II scores.

Figure 2 illustrates these patterns, highlighting how the direction of microbiota associations with dietary patterns aligns or contrasts with their associations with BDI‐II. Values of the coefficients and confidence intervals in Figure 2 can be found in Tables S3–S8.

### 2.6 Gut Microbiota Mediates the Beneficial Effect of Mediterranean Diet on Depression Symptomatology

The mediation analysis assessed whether gut microbiota mediated the association between dietary patterns and depressive symptoms, measured by BDI‐II. The results are expressed in Table 3, and the corresponding a‐path (dietary pattern → GMS) and b‐path (GMS → BDI‐II) estimates are reported in Table S9.

**TABLE 3:** Mediation analysis assessing gut microbiota as a mediator between dietary patterns and depressive symptoms, using longitudinal and change‐based models.

Dietary pattern	Indirect effect (ACME)	Direct effect (ADE)	Total effect (TE)	Proportion mediated (P.MED)
	β (95%CI) *p*‐value	β (95%CI) *p*‐value	β (95%CI) *p*‐value	% (95%CI) *p*‐value
Longitudinal models (dietary pattern → gut microbiota score → BDI‐II)				
erMEDAS	**−0.029 (−0.054, −0.006)** ***p* = 0.011**	**−0.139 (−0.259, −0.019)** ***p* = 0.022**	**−0.168 (−0.285, −0.052)** ***p* = 0.005**	**17 (3, 61.8)** ***p* = 0.016**
MEDAS	**−0.066 (−0.116, −0.018)** ***p* = 0.006**	−0.142 (−0.326, 0.046) *p* = 0.135	**−0.208 (−0.389, −0.026)** ***p* = 0.022**	**31.1 (5, 170.2)** ***p* = 0.027**
DASH	−0.021 (−0.096, 0.052) *p* = 0.582	**−0.091 (−0.175, ‐0.007)** ***p* = 0.031**	**−0.113 (−0.184, −0.043)** ***p* = 0.003**	19 (−67.6, 92.9) *p* = 0.579
HPDI	−0.012 (−0.065, 0.039) *p* = 0.648	−0.047 (−0.102, 0.01) *p* = 0.106	**−0.059 (−0.103, −0.015)** ***p* = 0.01**	20.8 (−115.7, 129.2) *p* = 0.643
WESTDIET	0.03 (−0.021, 0.081) *p* = 0.251	**0.061 (0.002, 0.119)** ***p* = 0.043**	**0.091 (0.042, 0.139)** ***p* < 0.001**	33.6 (−29.4, 97) *p* = 0.251
UPDI	−0.003 (−0.062, 0.054) *p* = 0.927	0.051 (−0.015, 0.117) *p* = 0.117	0.048 (−0.007, 0.102) *p* = 0.088	−1.8 (−406.6, 278.1) *p* = 0.971
Change‐based models (Δdietary pattern → Δgut microbiota score →ΔBDI‐II)				
erMEDAS	0.023 (−0.014, 0.061) *p* = 0.218	**−0.198 (−0.342, −0.057)** ***p* = 0.005**	**−0.175 (−0.316, −0.036)** ***p* = 0.012**	−12.7 (−65.3, 11.4) *p* = 0.228
MEDAS	0.024 (−0.032, 0.082) *p* = 0.39	−0.197 (−0.433, 0.037) p = 0.102	−0.173 (−0.405, 0.056) *p* = 0.139	−11.8 (−156.4, 99.8) *p* = 0.47
DASH	0.019 (−0.001, 0.041) *p* = 0.06	**−0.142 (−0.23, −0.055)** ***p* = 0.001**	**−0.124 (−0.208, −0.038)** ***p* = 0.003**	−14.8 (−59.1, 0.7) *p* = 0.061
HPDI	0.005 (−0.005, 0.017) *p* = 0.308	**−0.063 (−0.116, −0.009)** ***p* = 0.019**	**−0.057 (−0.109, −0.005)** ***p* = 0.029**	−8.6 (−68.2, 15.9) *p* = 0.328
WESTDIET	−0.009 (−0.024, 0.004) *p* = 0.164	**0.103 (0.044, 0.159)** ***p*<0.001**	**0.093 (0.036, 0.147)** ***p* = 0.002**	−9.8 (−38, 4.9) *p* = 0.166
UPDI	**−0.014 (−0.03, 0)** ***p* = 0.043**	**0.076 (0.009, 0.144)** ***p* = 0.027**	0.062 (−0.005, 0.128) *p* = 0.065	−20.8 (−152.1, 52.4) *p* = 0.106

*Note*: Longitudinal mediation analyses were performed with linear mixed‐effect models adjusted for visit, gender, age, study center, BMI, employment status, marital status, education level, alcohol consumption (g/day), hypertension prevalence, diabetes prevalence, intervention group, smoking status, antidepressant use, and participant ID as random intercept. Changes‐based mediation analyses were performed with linear models adjusted for baseline dietary pattern score, baseline gut microbiota score, baseline BDI‐II score, gender, age, study center, BMI, employment status, marital status, education level, changes in alcohol consumption (g/day), hypertension prevalence, diabetes prevalence, intervention group, smoking status, and antidepressant use. ACME represents the indirect effect of dietary patterns on depressive symptoms (BDI‐II) through gut microbiota, where gut microbiota is characterized by the gut microbiota score specific to each dietary pattern (dietary pattern → gut microbiota score → BDI‐II). ADE represents the direct association between dietary patterns and depressive symptoms (dietary pattern → BDI‐II) after accounting for the mediator. TE represents the total association between dietary patterns and depressive symptoms (dietary pattern → BDI‐II) before considering mediation. P.MED represents the proportion of the total effect mediated by gut microbiota. The gut microbiota score for each dietary pattern was derived based on taxa identified as associated with that dietary pattern. Estimates are presented as β (95% CI), *p*‐value. Bold values indicate statistical significance (*p* < 0.05).

Abbreviations: ACME, average causal mediation effect; ADE, average direct effect; BDI‐II, Beck Depression Inventory‐II; DASH, Dietary Approaches to Stop Hypertension; erMEDAS, Energy‐Reduced Mediterranean Diet Adherence Score; HPDI, Healthy Plant‐Based Diet Index; MEDAS, Mediterranean Diet Adherence Score; P.MED, proportion mediated; TE, total effect; UPDI, Unhealthy Plant‐Based Diet Index; WESTDIET, Western Diet Score.

**FIGURE 3:**
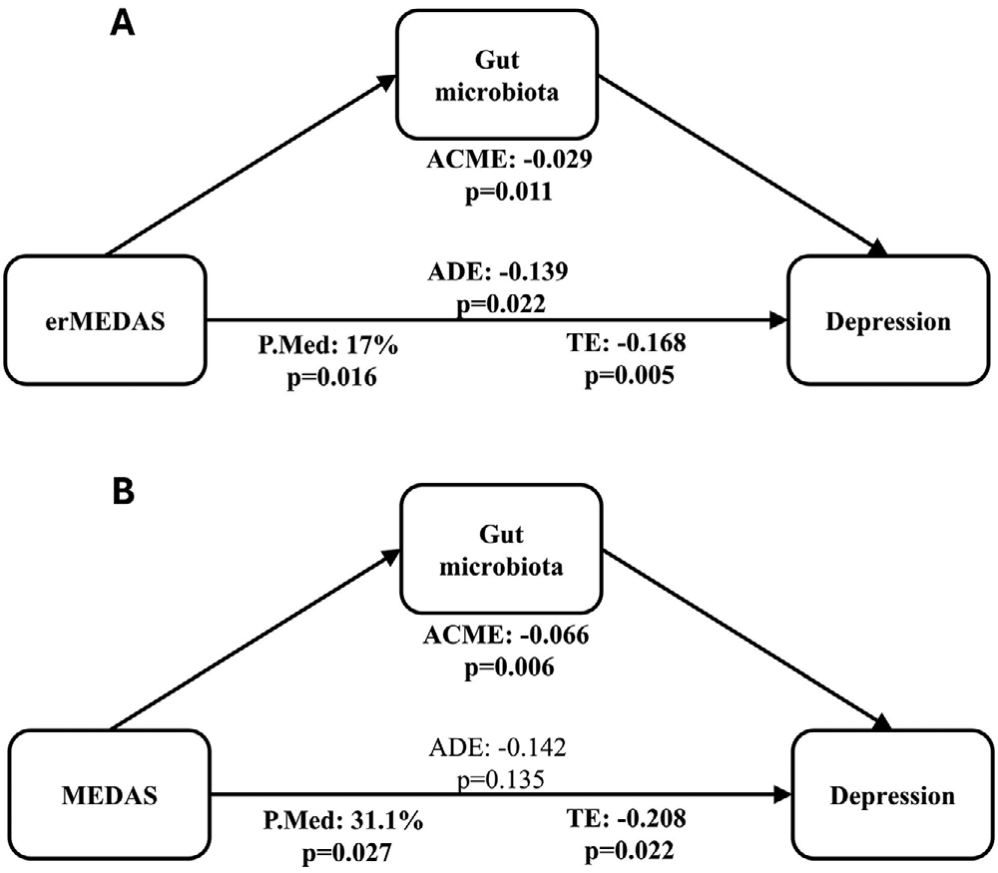
(A) Mediation analysis diagram of the effect of erMEDAS (Energy‐Reduced Mediterranean Diet Adherence Score) on depressive symptoms, with gut microbiota profile as mediator. (B) Mediation analysis diagram of the effect of MEDAS (Mediterranean Diet Adherence Score) on depressive symptoms, with gut microbiota profile as mediator. ACME, average causal mediation effect; ADE, average direct effect; P.MED, proportion mediated; TE, total effect.

All the dietary patterns were tested despite some nonsignificant direct associations. Among the tested dietary patterns, erMEDAS and MEDAS in the longitudinal models, and UPDI in the change‐based model, showed significant mediation effects through gut microbiota. For erMEDAS, the longitudinal model showed a significant indirect effect (ACME: –0.029, *p* = 0.011), with 17.0% of the total effect mediated by gut microbiota (*p* = 0.016); both the direct effect (ADE: –0.139, *p* = 0.022) and the total effect (TE: –0.168, *p* = 0.005) were also significant. For MEDAS, a significant indirect effect was observed (ACME: –0.066, *p* = 0.006), with 31.1% of the total effect mediated (P.Med: 31.1%, *p* = 0.027), while the total effect was significant (TE: –0.208, *p* = 0.022) but the direct effect was not (ADE: –0.142, *p* = 0.135) (Figure 3). A sensitivity analysis revealed that the significant indirect effect remained even when excluding participants who were receiving antidepressant treatment for erMEDAS (ACME: –0.027, *p* = 0.016) and MEDAS (ACME: –0.078, *p* = 0.001). For these two patterns, we also ran taxon‐level mediation and found that *Unclassified UCG‐10* mediates the beneficial effect of erMEDAS on depression symptomatology (ACME: −0.010, *p* = 0.012, ADE: −0.158, *p* = 0.020, TE: −0.167, *p* = 0.012, P.Med: 5.44%, *p* = 0.024). In the change‐based models (changes in dietary patterns, gut microbiota, and depression symptomatology), UPDI showed a significant indirect effect (ACME: –0.014, *p* = 0.043), although the total effect (TE: 0.062, *p* = 0.065) and the proportion mediated (P.Med: –20.8%, *p* = 0.106) did not reach significance.

## 3 Discussion

This study presents a comprehensive analysis of the associations between six dietary patterns, gut microbiota composition, and depressive symptomatology, using data from a large, well‐characterized cohort of older adults participating in the PREDIMED‐Plus trial. By integrating repeated measures of diet, microbiota, and mental health, this work provides novel insights into the potential mediating role of the gut microbiota in the diet–depression relationship. The study design allowed for longitudinal evaluations, as well as analyses of within‐subject changes over time. This multidimensional approach strengthens the interpretation of findings by capturing the dynamic interactions between dietary patterns, gut microbial ecology, and mental health outcomes. The identification of dietary pattern‐specific microbial signatures and their differential associations with depressive symptoms provides further evidence for the role of the gut–brain axis in mental health and underscores the relevance of dietary quality in modulating these pathways.

The overall findings of this study indicate that adherence to healthier dietary patterns is associated with lower depressive symptomatology, while less healthy patterns show the opposite trend, a result that is in line with previous evidence [17, 18, 19, 20]. The healthy dietary patterns analyzed were characterized by higher intakes of fruits and vegetables, legumes, nuts, whole grains, and fish, and lower intakes of red and processed meats, features that are consistent with anti‐inflammatory and antioxidant‐rich dietary profiles [21, 22, 23]. In contrast, unhealthy patterns were typically high in ultraprocessed foods, saturated fats, and refined sugars, and low in fiber, all of which have been associated with proinflammatory states [24, 25]. Current evidence suggests that chronic low‐grade inflammation, oxidative stress, impaired antioxidant defence mechanisms, and insulin resistance may contribute to the development of psychiatric disorders, including depression [26, 27]. Fruits and vegetables are rich in dietary fiber, phytochemicals, and micronutrients with antioxidant properties, which may help reduce oxidative stress and inflammation, thereby lowering the risk of depressive symptoms [28, 29]. Moreover, these healthy dietary patterns are associated with greater gut microbiota diversity and richness, which are considered hallmarks of a balanced and resilient microbial ecosystem [30, 31, 32, 33]. This is consistent with our findings, where adherence to healthier diets corresponded to higher alpha diversity metrics, including richness, evenness, and overall diversity, further supporting the relevance of gut microbial biodiversity in mental health [34, 35].

Most of the taxa linked to dietary patterns in our study fell within the *Clostridia* class, a metabolically versatile Firmicutes group known for anaerobic fermentation of complex substrates into short‐chain fatty acids and other bioactive compounds that modulate immunity, barrier integrity, and neural signaling [36]. Notably, several of these *Clostridia* show consistent associations with depressive symptoms: *Lachnospiraceae NK4A136 group* and *Eubacterium xylanophilum group* are depleted in depressed subjects, suggesting protective roles [37, 38], while *Hungatella* is enriched in individuals with higher symptom scores [39]. Our own data also demonstrate lower *Oscillospiraceae NK4A214* and *Christensenellaceae R‐7 groups* abundance among those with depression, reinforcing their potential involvement in gut–brain interactions [34], and lower *Subdoligranulum* and *Lachnospiraceae UCG‐001* levels have been linked to anxiety, cognitive decline, and depressive features in older adults [39].

The mediation analyses revealed that gut microbiota significantly mediates the relationship between Mediterranean dietary adherence and depressive symptoms. In the longitudinal models, both erMEDAS and MEDAS exhibited significant indirect effects, but erMEDAS showed partial mediation, with both indirect and direct effects significant, indicating that its energy‐reduction recommendations may not only influence depression via microbial pathways but also through other mechanisms. Although MEDAS and erMEDAS are closely related Mediterranean‐diet adherence measures, they differ in scope and thresholds: MEDAS comprises 14 items reflecting core Mediterranean features, whereas erMEDAS is a 17‐item energy‐restricted adaptation with stricter limits on energy‐dense foods and additional items targeting caloric reduction. This stronger, multifaceted mediation may stem from caloric restriction enhancing microbial diversity, metabolic function, and reducing inflammation, all factors known to affect mental health outcomes [40, 41, 42]. By contrast, MEDAS displayed a significant indirect effect alongside a nonsignificant direct effect, consistent with full mediation and suggesting its impact operates predominantly through changes in the microbiota. However, this specificity does not confer a greater overall benefit than erMEDAS, as partial mediation suggests erMEDAS's broader and more resilient influence. Taxon‐level mediation identified *Unclassified UCG‐10* as a significant mediator between erMEDAS and depression, aligning with the direction of the composite microbiota score. Given that UCG denotes an uncultured clade, this finding should be interpreted cautiously. In change‐based models, UPDI produced a significant indirect effect despite no total or direct effect, a suppressor pattern that implies microbial shifts from an unhealthy plant‐based diet may counterbalance expected mood outcomes. While these short‐term changes did not translate into overall symptom improvements, the significant ACME underscores the potential for gut microbiota to modulate depressive symptoms even under suboptimal dietary conditions, although longer follow‐up may be required to detect clinical effects.

This study has several limitations that should be acknowledged. First, reverse causation may be partly addressed by the use of longitudinal and change‐based models, as well as mediation analysis to explore potential causal pathways. However, due to the inherently observational nature of the study design, causal relationships between dietary patterns, gut microbiota, and depressive symptoms cannot be definitively established. Although longitudinal and change‐based models were adjusted for covariates, residual confounding cannot be entirely ruled out. Second, although dietary intake was collected using a validated, interviewer‐administered food frequency questionnaire conducted by trained dietitians, some degree of measurement error inherent to dietary assessment methods remains possible. Third, gut microbiota was characterized using 16S rRNA gene sequencing, which limits taxonomic resolution to the genus level and does not allow direct inference of microbial function or strain‐specific effects. Fourth, the study population consisted of older adults with overweight or obesity and metabolic syndrome risk factors, which may limit the generalizability of the findings to other populations. Fifth, while mediation analyses adjusted for a wide range of potential confounders, unmeasured variables, including psychosocial stressors, other medication use, and lifestyle factors, may still have influenced the results. Finally, although the longitudinal design strengthens the plausibility of mediation pathways, the 1‐year follow‐up period may not be sufficient to fully capture long‐term microbiota and mental health changes.

This study also has several important strengths. It is one of the few analyses to comprehensively integrate dietary pattern assessments, gut microbiota profiling, and depressive symptomatology within a large, multicenter, well‐characterized cohort of older adults at high cardiometabolic risk. The use of a validated, interviewer‐administered food frequency questionnaire by trained dietitians enhanced the accuracy of dietary intake measurements, while depressive symptoms were evaluated using the well‐established BDI‐II. The population consisted of community‐dwelling older adults, a group representing a growing and clinically relevant sector of the global population. Although the data were collected within the framework of a randomized controlled trial, the associations analyzed in this study reflect natural variations in dietary behaviors and microbiota composition within the intervention and control arms, thereby preserving relevance to real‐world settings. The application of both longitudinal and change‐based models, alongside mediation analyses, allowed for a nuanced understanding of the dynamic relationships between diet, gut microbiota, and mental health, while repeated microbiota measures, comprehensive covariate adjustments, and sensitivity analyses strengthened the robustness and interpretability of the results.

In conclusion, this study provides novel evidence linking dietary patterns to depressive symptomatology, with findings suggesting that gut microbiota may play a mediating role in this relationship in a cohort of community‐dwelling older adults. These findings support the potential role of dietary interventions targeting the gut microbiota as a complementary strategy for the prevention and management of depression. Future research should aim to confirm these findings in other populations and explore underlying microbial mechanisms in greater functional detail.

## 4 Materials and Methods

### 4.1 Study Design

This longitudinal observational analysis took place within the context of the PREDIMED‐Plus project, a multicenter randomized controlled trial currently underway at 23 locations across Spain. The study is designed to examine the effects of an intervention that integrates a reduced‐calorie Mediterranean diet, increased physical activity, and motivational support on the primary prevention of cardiovascular disease. Additional information about the study protocol is available at https://www.predimedplus.com/ and in prior publications [43, 44]. All participating centers received ethical approval, and written informed consent was obtained from every participant. The trial was initially registered in 2014 at the International Standard Randomized Controlled Trial registry [www.isrctn.com/ISRCTN89898870].

### 4.2 Participants

**FIGURE 4:**
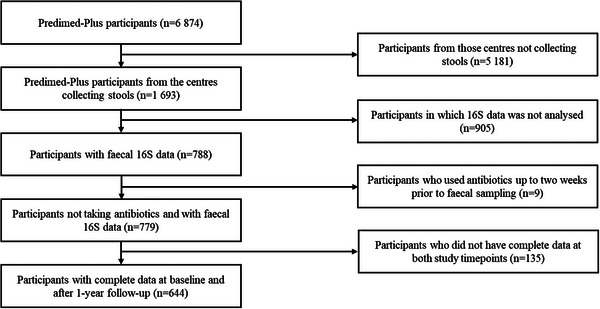
Flowchart of the study population. Abbreviations: 16S, 16S ribosomal RNA gene sequencing; PREDIMED‐Plus, PREvención con Dieta Mediterránea‐Plus.

The participants included adult men aged 55–75 and adult women aged 60–75, who were classified as overweight or obese, with a body mass index (BMI) between 27 and 40 kg/m^2^, suffering from metabolic syndrome due to meeting a minimum of three diagnostic criteria for said syndrome [45]. Individuals with a prior history of cardiovascular disease or major depressive disorder at the start of the study were not eligible for participation. Between October 2013 and December 2016, a total of 6,874 qualified individuals were randomized in equal numbers to either the intervention group or a control group, the latter receiving standard care along with general guidance on following a traditional Mediterranean diet without caloric restriction. For the current analysis, data were drawn from a subset of participants who had gut microbiota assessments conducted at four of the 23 participating centers. We additionally excluded those participants who were taking antibiotics at least 2 weeks before the stool collection (*n* = 9) and those who had missing data at any of the sociodemographic and lifestyle factors (*n* = 4). Therefore, data from 644 individuals (mean age 65 ± 4.9 years, 47% women) presenting available gut microbiota measures and complete data at baseline and 1 year thereafter were considered (Figure 4).

### 4.3 Exposure: Dietary Pattern Scores

Dietary intake data were collected using a validated food frequency questionnaire (FFQ) that included portion sizes and nine categories of consumption frequency, ranging from “never or almost never” to “six or more times per day” [46]. To estimate energy and nutrient intakes, reported frequencies were multiplied by the standard portion [47]. Nutrient values were calculated using data from Spanish food composition tables [47].

A total of ten dietary pattern adherence scores, as continuous, were computed, and six were selected based on their significant associations with depression symptomatology in preliminary analyses. Four of these dietary pattern scores, including the Healthy Plant‐Based Diet Index (HPDI), Unhealthy Plant‐Based Diet Index (UPDI), Dietary Approaches to Stop Hypertension (DASH), and Western Diet (WESTDIET), were derived from responses to the 143‐item food frequency questionnaire. The energy‐reduced Mediterranean Diet Adherence Score (erMEDAS) was calculated separately based on a 17‐item Mediterranean diet adherence screener, which was administered through in‐person interviews conducted by trained personnel [48]. The Mediterranean Diet Adherence Score (MEDAS) was estimated using information from both the FFQ and corresponding items from the 17‐point screener.

Details on dietary patterns and their scoring criteria are provided in the Supplementary Materials and can also be found elsewhere [49].

### 4.4 Outcome: Depression Symptomatology

Depressive symptomatology was measured using the BDI‐II, a widely used and validated self‐report instrument for assessing the severity of depressive symptoms in adolescents and adults [50]. The questionnaire consists of 21 items, each rated on a 4‐point scale based on how participants have felt during the previous 2 weeks, yielding a total score ranging from 0 to 63. The BDI‐II captures a broad range of symptom domains, including cognitive, affective, somatic, and motivational components. Total scores are typically interpreted as follows: 0–13 (minimal), 14–19 (mild), 20–28 (moderate), and 29–63 (severe) depressive symptoms [50]. The instrument has shown excellent internal consistency, with Cronbach's alpha values frequently above 0.90, as well as strong test–retest reliability and construct validity, demonstrated through correlations with clinical diagnoses and other standardized depression scales [50]. In the present study, BDI‐II scores were analyzed as a continuous variable to assess associations with dietary patterns and gut microbiota, with results interpreted per unit increase in total score.

### 4.5 Stool Samples Collection, DNA Extraction, 16S rRNA Gene Amplicon Sequencing, and Taxonomy Assignment

Faecal specimens were self‐collected by participants at home and immediately frozen until transported to the laboratory. Upon arrival, samples were subdivided into 250 mg portions and stored at −80°C for subsequent analysis. Extraction of microbial DNA was performed using the QIAmp PowerFecal DNA kit (Qiagen, Hilden, Germany), following the protocol provided by the manufacturer. Before extraction, samples underwent a mechanical lysis step for five minutes using the FastPrep‐24 5G Homogenizer (MP Biomedicals, USA). The resulting DNA was then stored at −20°C. Concentration and purity of the extracted DNA were quantified using the Qubit 2.0 Fluorometer and dsDNA BR assay kit (Thermo Fisher Scientific, USA).

For amplicon sequencing, the V4 region of the 16S rRNA gene was targeted using primers 515F and 806R, with PCR reactions run in triplicate for each sample. Each 35 µL reaction mixture included 0.7 µL of uniquely barcoded primers (10 µM), 0.7 µL dNTP mix, 0.35 µL Phusion Green Hot Start II high‐fidelity DNA polymerase (2 U/µL; Thermo Scientific, the Netherlands), 7 µL of 5× Phusion Green HF buffer, and 25.5 µL DNase/RNase‐free water (Promega, USA). Amplification conditions involved an initial denaturation at 98°C for 30 s, followed by 25 cycles consisting of 10 s at 98°C, 10 s at 50°C, and 10 s at 72°C, with a final extension at 72°C for 7 min. PCR product size (∼290 bp) was verified on 1% agarose gel, and purification was carried out using the CleanPCR kit (CleanNA, the Netherlands). Concentration of purified PCR amplicons was assessed with the Qubit dsDNA BR assay kit, and 200 ng of DNA per sample was used for library preparation. Sequencing was performed using the Illumina Novaseq platform (as described in previously published works) [34, 51].

To ensure quality control, positive controls (artificial mock communities of known composition) and technical replicates (seven duplicated fecal samples) were included. Negative controls, such as DNA extraction blanks and nuclease‐free water, were also processed to monitor for contamination. Amplicon sequence variants (ASVs) were determined using the DADA2 pipeline (v 1.16), with taxonomic classification based on the Silva v138.1 database. Sequencing generated a median of 1,120,481 paired‐end reads per sample (IQR: [379,416–1,645,145]). After filtering, denoising, and removal of chimeric reads (11% of total), an average of 948,831 nonchimeric reads per sample (median: 969,828, IQR: [334,276–1,415,867]) was retained. All samples surpassed the minimum sequencing depth threshold of 10,000 reads, and thus none were excluded due to low read count.

### 4.6 Covariate Assessments

Information on participants’ age, sex, education, marital status, and smoking habits was collected using questionnaires. Weight and height were measured with calibrated scales and stadiometers. BMI was calculated by dividing weight in kilograms by height in meters squared. Physical activity was assessed with the REGICOR Short Physical Activity Questionnaire, which is validated for adults and based on the Minnesota Leisure Time Physical Activity Questionnaire (MLTPAQ) [52]. Data on health conditions like type 2 diabetes, high blood pressure, and antidepressant use came from either self‐report or medical records.

### 4.7 Statistical Analyses

Main analyses were conducted in the evaluable population, including all participants with available exposure and outcome data at baseline and 1‐year follow‐up. Baseline characteristics of the study cohort were described for the overall sample, with continuous variables presented as medians and interquartile ranges (IQRs) and categorical variables as frequencies and percentages. Descriptive comparisons were performed using the Kruskal–Wallis rank‐sum test for continuous variables, and either Pearson's chi‐squared test or Fisher's exact test for categorical variables, as appropriate.

To assess the association between dietary pattern scores and BDI‐II, linear mixed‐effects models were fitted using data from both baseline and 1‐year follow‐up. Participant ID was included as a random intercept to account for repeated measures. Additionally, linear models were used to evaluate the association between changes in dietary pattern scores and changes in BDI‐II.

The LMM analyses were conducted using three sequential models. Model 1 included only a random intercept for participants. Model 2 adjusted for visit, sex, age, and study center. Model 3 further adjusted for BMI, employment status, marital status, education level, alcohol consumption (g/day), hypertension prevalence, diabetes prevalence, intervention group, smoking status, and antidepressant use. For the linear models assessing the association between changes in dietary pattern scores and changes in BDI‐II, Model 1 included the change in dietary pattern score as exposure, adjusted for its baseline level and baseline BDI‐II. Model 2 additionally adjusted for sex and age. Model 3 further adjusted for intervention group, study center, BMI, employment status, marital status, education level, changes in alcohol consumption (g/day), hypertension prevalence, diabetes prevalence, smoking status, and antidepressant use. In this cohort, all participants with hypertension/diabetes are under standard pharmacologic treatment; thus, the hypertension and diabetes covariates account for both diagnosis and treatment. Only the dietary patterns that were significantly associated with depression symptomatology were used in the subsequent analyses. As a sensitivity analysis, effect modification by sex was evaluated by adding a diet × sex term and comparing models via a likelihood ratio test across the six indices

Gut microbiota sequencing data at the amplicon sequent variant (ASV) level were aggregated to the genus level. Taxa were filtered using a detection threshold of 1% and a prevalence threshold of 10%, based on relative abundance. Relative abundances were calculated from absolute counts, and centered log‐ratio (CLR) transformations were applied to the relative abundance data after adding a pseudocount to handle zero values. To assess within‐sample diversity (alpha diversity), the Chao1 (richness), Shannon (diversity), and inverse Simpson (evenness) indices were calculated using absolute counts of ASVs. Between‐sample diversity (beta diversity) was calculated using Bray–Curtis distances on total sum scaled (TSS) transformed counts at genus level with a filtering threshold of 10% prevalence and a detection level of 0.1%. To explore additional associations, we assessed the relationship between dietary pattern scores and alpha diversity indices (Chao1, Shannon, and inverse Simpson). These associations were evaluated using LMMs, including participant ID as a random intercept to account for repeated measures at baseline and 1‐year follow‐up. Models were adjusted for the same covariates described in the previous analyses.

We employed a linear mixed‐effects model with least absolute shrinkage and selection operator (LMM‐LASSO) penalty to identify microbial taxa robustly linked with longitudinal dietary pattern scores. This approach was used to construct a gut microbiota score specific to each dietary pattern, with the aim of capturing diet‐related microbial signatures for use in subsequent mediation analyses evaluating the potential role of gut microbiota in the association between diet and depressive symptomatology. In these models, dietary pattern scores were treated as targets, and the CLR microbial taxa counts were used as predictors. The LMM‐LASSO approach simultaneously accounted for repeated measures (baseline and 1‐year follow‐up) through subject‐specific random intercepts and applied L1 regularization to perform variable selection, shrinking unstable or collinear taxa to zero and retaining those with consistent multivariate associations across timepoints. A 10‐fold cross‐validation procedure was implemented, where 90% of the data was used for training and the remaining 10% for validation. We explored 100 values of the regularization parameter (*λ*) between 0.01 and 1000, and the *λ* that minimized the Akaike information criterion (AIC) in each fold was selected for the final model. Taxa consistently selected across all 10 cross‐validation folds were considered as associated with its dietary pattern.

The final microbiota score for each dietary pattern was computed by aggregating the alpha diversity indices (Chao1, Shannon, and inverse Simpson), the first two principal components of beta diversity (PC1 and PC2), and the selected taxa identified for each dietary pattern through LMM‐LASSO. With this approach, we constructed a gut microbiota score for each dietary pattern that reflects multiple dimensions of gut microbiota structure, including alpha and beta diversity, specific taxonomic composition, and longitudinal consistency captured through the mixed‐effects framework. In the microbiome domain, analogous score‑building frameworks based on penalized regression have been applied [53, 54]. Further details on how the gut microbiota score is calculated can be found in the supplementary materials.

Mediation analyses were conducted to evaluate whether the gut microbiota score of each dietary pattern mediated the association between its dietary pattern score and depressive symptomatology. For each dietary pattern, a mediation analysis was performed using BDI‐II as the outcome, the dietary pattern score as the exposure, and the gut microbiota score of the same pattern as the mediator, adjusting for visit (baseline, 1 year), sex (female, male), age (years), study recruitment center (Reus, Valencia, Alicante, Barcelona), BMI (kg/m^2^), employment status (active, unemployed, retired), marital status (married, other), education level (primary or less, secondary, higher education), alcohol consumption (g/day), hypertension prevalence (yes, no), diabetes prevalence (yes, no), intervention group (control, intervention), smoking status (current, former, never smoker), antidepressant use (yes, no) and participant ID specified as the grouping variable. The analysis was performed using the “mediate” function from the mediation R package, with 5000 simulations to estimate the confidence intervals for the mediation effects and assess statistical significance. The metrics obtained were the average causal mediation effect (ACME), which represents the indirect effect of dietary patterns on BDI‐II through gut microbiota; the average direct effect (ADE), which represents the direct effect of dietary patterns on BDI‐II, independent of the gut microbiota; and the total effect (TE), which is the sum of ACME and ADE, reflecting the overall association between dietary patterns and BDI‐II. Additionally, the proportion mediated (P.Med) quantifies the percentage of the total effect that is explained by the indirect pathway through gut microbiota. For dietary patterns with significant mediation by the microbiota score, we conducted exploratory taxon‐level mediation, testing each selected taxon as the mediator using the same procedure as above. All statistical analyses were performed using R (version 4.3.2).

## Author Contributions

All the principal PREDIMED‐Plus investigators contributed to the study concept and design and to data extraction from the PREDIMED‐Plus participants. A.H.‐C., JF.G.‐G., and J.S.‐S. contributed to the concept and design of the present study. A.H.‐C. wrote the first draft and performed the statistical analyses under the supervision of JF.G.‐G. A.H.‐C., JF.G.‐G., and J.S.‐S. are the guarantors of this work and, as such, had full access to all the data in the study and take responsibility for the integrity of the data and the accuracy of the data analysis. All authors reviewed the manuscript for important intellectual content and approved the final version to be published.

## Funding

This work was supported by the official Spanish Institutions for funding scientific biomedical research, CIBER Fisiopatología de la Obesidad y Nutrición (CIBEROBN) and Instituto de Salud Carlos III (ISCIII), through the Fondo de Investigación para la Salud (FIS), which is co‐funded by the European Regional Development Fund (six coordinated FIS projects leaded by J.S.‐S. and J.Vi., including the following projects: PI13/00233, PI13/00728, PI13/00462, PI14/01206, PI14/ 00696, PI16/00533, PI16/00366, PI16/00501, PI17/01441, PI17/00855, PI19/00017, PI19/00781, PI19/00576, PI20/00557, PI21/0046; the Especial Action Project entitled: Implementación y evaluación de una intervención intensiva sobre la actividad física Cohorte PREDIMED‐Plus grant to J.S.‐S; the Recercaixa (number 2013ACUP00194) grant to J.S.‐S.; grants from the Consejería de Salud de la Junta de Andalucía (PI0458/2013, PS0358/2016, PI0137/2018); the PROMETEO/ 2017/017 and PROMETEO/2021/21 grants from the Conselleria de Innovación, Universidades, Ciencia y Sociedad Digital from the Generalitat Valenciana; and by NIH grant R01DK127601. This research was also partially funded by the Eat2beNICE/H2020‐SFS‐2016‐2 EU‐H2020 European grant and the Horizon 2020 PRIME study (Prevention and Remediation of Insulin Multimorbidity in Europe; grant agreement #847879). J.S.‐S., the senior author of this paper, was partially supported by ICREA under the ICREA Academia program. A.H.‐C. is supported by a predoctoral grant from Martí Franquès—INVESTIGO research fellowship funded and supported by NextGenerationEU, Servicio Público de Empleo Estatal, and Universitat Rovira i Virgili (2022PMF‐INV‐01). I.M.‐I. was supported by a Miguel Servet type II grant from Instituto de Salud Carlos III, Madrid, Spain (CPII21/00013). J.N. is supported by a predoctoral grant from Ministerio de Ciencia, Innovación y Universidades (FPU 20/00385). None of the funding sources took part in the design, collection, analysis, interpretation of the data, writing the report, or in the decision to submit the manuscript for publication.

## Conflicts of Interest

The authors declare no conflicts of interest.

## Ethics Statement

All participating centers received ethical approval, and written informed consent was obtained from every participant. The trial was initially registered in 2014 at the International Standard Randomized Controlled Trial registry [www.isrctn.com/ISRCTN89898870].

## Supporting information




**Table S1**: Baseline characteristics of the population included in the present study, the total population of the four recruiting centers included in this study, and all the PREDIMED‐Plus study population.
**Table S2**. Baseline values and 1‐year changes in BDI‐II, dietary pattern scores, and gut microbiota scores for each dietary pattern in the study population.
**Table S3**: Genera that better predict adherence to the erMEDAS dietary pattern in the study population according to LMM‐LASSO. The table presents the beta coefficients (B) and 95% confidence intervals (95% CI) from linear regression models assessing the association between the erMEDAS score and the relative abundance of each genus. For reference, associations with depressive symptoms measured by the BDI‐II score are also shown. Positive beta values indicate a direct association, while negative values reflect an inverse association.
**Table S4**: Genera that better predict adherence to the MEDAS dietary pattern in the study population according to LMM‐LASSO. The table presents the beta coefficients (B) and 95% confidence intervals (95% CI) from linear regression models assessing the association between the MEDAS score and the relative abundance of each genus. For reference, associations with depressive symptoms measured by the BDI‐II score are also shown. Positive beta values indicate a direct association, while negative values reflect an inverse association.
**Table S5**: Genera that better predict adherence to the DASH dietary pattern in the study population according to LMM‐LASSO. The table presents the beta coefficients (B) and 95% confidence intervals (95% CI) from linear regression models assessing the association between the DASH score and the relative abundance of each genus. For reference, associations with depressive symptoms measured by the BDI‐II score are also shown. Positive beta values indicate a direct association, while negative values reflect an inverse association.
**Table S6**: Genera that better predict adherence to the HPDI dietary pattern in the study population according to LMM‐LASSO. The table presents the beta coefficients (B) and 95% confidence intervals (95% CI) from linear regression models assessing the association between the HPDI score and the relative abundance of each genus. For reference, associations with depressive symptoms measured by the BDI‐II score are also shown. Positive beta values indicate a direct association, while negative values reflect an inverse association.
**Table S7**: Genera that better predict adherence to the WESTDIET dietary pattern in the study population according to LMM‐LASSO. The table presents the beta coefficients (B) and 95% confidence intervals (95% CI) from linear regression models assessing the association between the WESTDIET score and the relative abundance of each genus. For reference, associations with depressive symptoms measured by the BDI‐II score are also shown. Positive beta values indicate a direct association, while negative values reflect an inverse association.
**Table 8**: Genera that better predict adherence to the UPDI dietary pattern in the study population according to LMM‐LASSO. The table presents the beta coefficients (B) and 95% confidence intervals (95% CI) from linear regression models assessing the association between the UPDI score and the relative abundance of each genus. For reference, associations with depressive symptoms measured by the BDI‐II score are also shown. Positive beta values indicate a direct association, while negative values reflect an inverse association.
**Table 9**: Estimates for the mediation paths (a: Diet → GMS; b: GMS → BDI‐II) for both longitudinal associations and between their changes after 1 year.
**Figure S1**. LMM‐LASSO coefficient paths (left) and *λ* selection curves (AIC vs. log₁₀(*λ*), right) for the erMEDAS pattern across 10 cross‐validation folds. Red vertical lines indicate the AIC‐optimal *λ* in each fold.
**Figure S2**. LMM‐LASSO coefficient paths (left) and *λ* selection curves (AIC vs. log₁₀(*λ*), right) for the MEDAS pattern across 10 cross‐validation folds. Red vertical lines indicate the AIC‐optimal *λ* in each fold.
**Figure S3**. LMM‐LASSO coefficient paths (left) and *λ* selection curves (AIC vs. log₁₀(*λ*), right) for the DASH pattern across 10 cross‐validation folds. Red vertical lines indicate the AIC‐optimal *λ* in each fold.
**Figure S4**. LMM‐LASSO coefficient paths (left) and *λ* selection curves (AIC vs. log₁₀(*λ*), right) for the HPDI pattern across 10 cross‐validation folds. Red vertical lines indicate the AIC‐optimal *λ* in each fold.
**Figure S5**. LMM‐LASSO coefficient paths (left) and *λ* selection curves (AIC vs. log₁₀(*λ*), right) for the WESTDIET pattern across 10 cross‐validation folds. Red vertical lines indicate the AIC‐optimal *λ* in each fold.
**Figure S6**. LMM‐LASSO coefficient paths (left) and *λ* selection curves (AIC vs. log₁₀(*λ*), right) for the UPDI pattern across 10 cross‐validation folds. Red vertical lines indicate the AIC‐optimal *λ* in each fold.

## Data Availability

The datasets generated and analyzed during the current study are not publicly available due to data regulations and for ethical reasons, considering that this information might compromise research participants’ consent, because our participants only gave their consent for the use of their data by the original team of investigators. However, collaboration for data analyses can be requested by sending a letter to the PREDIMED‐Plus steering Committee (predimed_plus_scom-mittee@googlegroups.com). The request will then be passed to all the members of the PREDIMED‐Plus Steering Committee for deliberation.
