# Supplementary material for: The Gut Microbiota as a Mediator in the Relationship Between Dietary Patterns and Depression
Source: MedComm (2020). 2026 Jan 20;7(2):e70562. doi: 10.1002/mco2.70562 (PMC12820420; doi:10.1002/mco2.70562)
Supplement: Supplementary file 1 — Table S1: Baseline characteristics of the population included in the present study, the total population of the four recruiting centers included in this study, and all the PREDIMED‐Plus study population. Table S2. Baseline values and 1‐year changes in BDI‐II, dietary pattern scores, and gut microbiota scores for each dietary pattern in the study population. Table S3: Genera that better predict adherence to the erMEDAS dietary pattern in the study population according to LMM‐LASSO. The table presents the beta coefficients (B) and 95% confidence intervals (95% CI) from linear regression models assessing the association between the erMEDAS score and the relative abundance of each genus. For reference, associations with depressive symptoms measured by the BDI‐II score are also shown. Positive beta values indicate a direct association, while negative values reflect an inverse association. Table S4: Genera that better predict adherence to the MEDAS dietary pattern in the study population according to LMM‐LASSO. The table presents the beta coefficients (B) and 95% confidence intervals (95% CI) from linear regression models assessing the association between the MEDAS score and the relative abundance of each genus. For reference, associations with depressive symptoms measured by the BDI‐II score are also shown. Positive beta values indicate a direct association, while negative values reflect an inverse association. Table S5: Genera that better predict adherence to the DASH dietary pattern in the study population according to LMM‐LASSO. The table presents the beta coefficients (B) and 95% confidence intervals (95% CI) from linear regression models assessing the association between the DASH score and the relative abundance of each genus. For reference, associations with depressive symptoms measured by the BDI‐II score are also shown. Positive beta values indicate a direct association, while negative values reflect an inverse association. Table S6: Genera that better predict ad [file MCO2-7-e70562-s001.docx]

**Title: The gut microbiota as a mediator in the relationship between dietary patterns and depression.**

**Supplementary Materials**

**Description of the dietary patterns.**

Plant-based diets, which emphasize higher intake of plant-derived foods relative to animal-based products, have been linked to reduced cardiovascular disease risk.^1^ In this study, both the Healthy and Unhealthy Plant-Based Diet Indices (HPDI and UPDI) were calculated, with adherence scores ranging from 18 to 90 points.^2^ The Mediterranean diet, widely associated with beneficial health outcomes, particularly cardiovascular protection,^3^ was assessed in two forms: the energy-restricted version, scored from 0 to 17, based on a 17-item Mediterranean Diet Adherence Screener (erMEDAS) administered via face-to-face interviews; and the standard version, scored from 0 to 14, based on the validated 14-item Mediterranean Diet Adherence Screener (MEDAS).^4,5^ The Dietary Approaches to Stop Hypertension (DASH) diet, originally designed to prevent and control hypertension, was scored from 0 to 40 points.^6^ Lastly, the Western diet (WESTDIET), characterized by high intake of red and processed meats, fried foods, and low consumption of fruits, vegetables, and fish, has been linked to various adverse health outcomes.^7,8^ Higher scores denote higher adherence to the dietary pattern. Adherence to these dietary patterns was evaluated to comprehensively explore potential associations between dietary habits and depressive symptomatology, as well as to identify gut microbiota profiles associated with the dietary pattern significantly associated with depression symptomatology.

1. Gan, Z. H., Cheong, H. C., Tu, Y. K. & Kuo, P. H. Association between Plant-Based Dietary Patterns and Risk of Cardiovascular Disease: A Systematic Review and Meta-Analysis of Prospective Cohort Studies. *Nutrients* **13**, (2021).

2. Satija, A. *et al.* Healthful and Unhealthful Plant-Based Diets and the Risk of Coronary Heart Disease in U.S. Adults. *J. Am. Coll. Cardiol.* **70**, 411–422 (2017).

3. Becerra-Tomás, N. *et al.* Mediterranean diet, cardiovascular disease and mortality in diabetes: A systematic review and meta-analysis of prospective cohort studies and randomized clinical trials. *Crit. Rev. Food Sci. Nutr.* **60**, 1207–1227 (2020).

4. García-Conesa, M. T. *et al.* Exploring the Validity of the 14-Item Mediterranean Diet Adherence Screener (MEDAS): A Cross-National Study in Seven European Countries around the Mediterranean Region. *Nutrients* **12**, 1–18 (2020).

5. Schröder, H. *et al.* A short screener is valid for assessing Mediterranean diet adherence among older Spanish men and women. *J. Nutr.* **141**, 1140–1145 (2011).

6. Fung, T. T. *et al.* Adherence to a DASH-style diet and risk of coronary heart disease and stroke in women. *Arch. Intern. Med.* **168**, 713–720 (2008).

7. Cordain, L. *et al.* Origins and evolution of the Western diet: health implications for the 21st century. *Am. J. Clin. Nutr.* **81**, 341–354 (2005).

8. Uribarri, J. *et al.* Advanced glycation end products in foods and a practical guide to their reduction in the diet. *J. Am. Diet. Assoc.* **110**, (2010).

**Procedure to create the gut microbiota score of each dietary pattern:**

Taxa (feature) selection via LMM-LASSO:

$\left( \hat{\alpha},\hat{\beta},\hat{\theta} \right)=\arg\max_{\alpha,\beta,\theta} \left\{ \mathcal{l}\left( \alpha,\beta,\theta;D \right)-\lambda\sum_{k=1}^{K} \left| \beta_{k} \right| \right\}, \theta=\left( \sigma_{u}^{2},\sigma^{2} \right)$. (1)

Where: $D$ denotes the observed data to penalize (all y_it_, and participant IDs); $\mathcal{l}\left( \alpha,\beta,\theta;D \right)$ is the mixed-model log-likelihood of the data given parameters; α is the unpenalized global intercept; $\beta=(\beta_{1},\ldots,\beta_{K})$ are fixed-effect coefficients for K predictors (e.g., genus abundances $G_{kit}$); λ ≥ 0 controls the L1​ (LASSO) penalty that shrinks $\sum_{k=1}^{K} \left| \beta_{k} \right|$ and can set some $\beta_{K}$ to zero. $\theta=\left( \sigma_{u}^{2},\sigma^{2} \right)$ collects the variance components, where $\sigma_{u}^{2}$ is the between-participant variance for the random intercepts and $\sigma^{2}$ is the residual (within-participant) variance; indices are$i$ for participants, $t$ for repeated measurements, and $k=1,\ldots,K$ for predictors. Maximizing the expression chooses $\left( \hat{\alpha},\hat{\beta},\hat{\theta} \right)$ that best fit the data (in this case, that best predicts the target, which is the dietary pattern score) while performing variable selection on the fixed effects, with the participant-specific random intercepts accounted for through $\sigma_{u}^{2}$ rather than being L1-penalized.

Dietary pattern gut microbiota score (GMS) calculation:

${GMS}_{it}=\alpha+\delta_{1}\text{C}_{\text{it}}+\delta_{2}\text{S}_{\text{it}}+\delta_{3}\text{I}\text{S}_{\text{it}}+\delta_{4}\text{PC}\text{1}_{\text{it}}+\delta_{5}\text{PC}\text{2}_{\text{it}}+\sum_{k=1}^{K} \beta_{k}G_{kit}+u_{i}+\varepsilon_{it}$(2)

$u_{i}\sim\mathcal{N}\left( 0,\sigma_{u}^{2} \right),\varepsilon_{it}\sim\mathcal{N}\left( 0,\sigma^{2} \right)$.

Where**:** ${GMS}_{it}$​ is the gut microbiota dietary pattern score for participant $i$ at time $t$; $i$ indexes participants and $t$ indexes repeated measurements; $\text{C}_{\text{it}}$ is the Chao1 alpha diversity value at time $t$ for participant $i$, ; $S_{\text{it}}$ is the Shannon alpha diversity value at time $t$ for participant $i$, ; ${Is}_{\text{it}}$ is the Inverse Simpson alpha diversity value at time $t$ for participant $i$, $\text{PC1}_{\text{it}}$ is the first principal component of beta diversity at time $t$ for participant $i$, $\text{PC2}_{\text{it}}$ is the second principal component of beta diversity at time $t$ for participant $i$, $G_{kit}$​ is the abundance of bacterial genus $k$ at time $t$ for participant $i$; $k=1,\ldots,K$ indexes genera; $\beta_{k}$​ are fixed-effect coefficients (estimated with an L1/LASSO penalty; the intercept $\alpha$ is not penalized); $\delta_{1,...,5}$ are the fixed-effect coefficients for alpha and beta diversity metrics, $u_{i}\sim\mathcal{N}\left( 0,\sigma_{u}^{2} \right)$ is the participant-specific random intercept; $\varepsilon_{it}\sim\mathcal{N}\left( 0,\sigma^{2} \right)$ is the residual error; $\sigma_{u}^{2}$​ is the between-participant variance and $\sigma^{2}$ is the within-participant (residual) variance.

| **Supplementary table S1:** Baseline characteristics of the population included in the present study, the total population of the four recruiting centres included in this study and all the PREDIMED-Plus study population | | | |
| --- | --- | --- | --- |
|  | **Study cohort** | **Population of the centres included in this study** | **Predimed-Plus cohort** |
| **Characteristic** | **N = 644**^1^ | **N = 1,693**^1^ | **N = 6,874**^1^ |
| BDI-II | 7 (3, 12) | 7 (3, 13) | 7 (3, 12) |
| **Sociodemographic variables** | | | |
| Female | 304 (47%) | 853 (51%) | 3,290 (49%) |
| Age | 65 (61, 68) | 65 (61, 69) | 65 (61, 69) |
| BMI | 32.5 (30.0, 35.5) | 32.3 (30.0, 35.4) | 32.2 (29.8, 34.9) |
| Intervention Group | 328 (51%) | 832 (49%) | 3,350 (49%) |
| Civil Status |  |  |  |
| Single or Separated | 89 (14%) | 202 (12%) | 882 (13%) |
| Married | 496 (77%) | 1,306 (78%) | 5,180 (77%) |
| Widowed | 59 (9.2%) | 173 (10%) | 704 (10%) |
| Religious | 0 (0%) | 1 (<0.1%) | 3 (<0.1%) |
| Education |  |  |  |
| Higher Education | 120 (19%) | 356 (21%) | 1,490 (22%) |
| Secondary School | 182 (28%) | 467 (28%) | 1,956 (29%) |
| Primary School or less | 342 (53%) | 859 (51%) | 3,323 (49%) |
| Employment |  |  |  |
| Active | 130 (20%) | 352 (21%) | 1,402 (21%) |
| Unemployed | 56 (8.7%) | 135 (8.0%) | 571 (8.4%) |
| Housekeeper | 77 (12%) | 210 (12%) | 1,002 (15%) |
| Student | 0 (0%) | 0 (0%) | 3 (<0.1%) |
| Retired | 381 (59%) | 985 (59%) | 3,791 (56%) |
| **Lifestyle factors and medication use** | | | |
| Alcohol consuption | 5 (1, 15) | 5 (1, 14) | 5 (1, 15) |
| Smoking status |  |  |  |
| Active smoker | 83 (13%) | 204 (12%) | 844 (12%) |
| Former smoker | 250 (39%) | 705 (42%) | 2,925 (43%) |
| Never smoked | 311 (48%) | 773 (46%) | 3,000 (44%) |
| **Dietary patterns score** | | | |
| erMEDAS | 8 (6, 10) | 8 (6, 10) | 8 (7, 10) |
| MEDAS | 8 (7, 10) | 8 (7, 9) | 8 (7, 9) |
| DASH | 24 (20, 27) | 24 (20, 27) | 24 (21, 28) |
| HPDI | 54 (49, 60) | 53 (48, 59) | 54 (49, 59) |
| WESTDIET | 36 (31, 40) | 36 (31, 41) | 36 (31, 41) |
| UPDI | 55 (50, 60) | 54 (49, 59) | 54 (49, 59) |
| ^1^n (%); Median (IQR). **Abbreviations**: BDI-II, Beck Depression Inventory-II; DASH, Dietary Approaches to Stop Hypertension; erMEDAS, Energy-Adjusted Mediterranean Diet Adherence Screener; HPDI, Healthful Plant-Based Diet Index; MEDAS, Mediterranean Diet Adherence Screener; UPDI, Unhealthful Plant-Based Diet Index; WESTDIET, Western Diet Index. | | | |

| **Supplementary Table S2.** Baseline values and 1-year changes in BDI-II, dietary pattern scores, and gut microbiota scores for each dietary pattern in the study population. | | |
| --- | --- | --- |
|  | **Baseline** | **1-year changes** |
| **Characteristic** | **N = 644**^1^ | |
| BDI-II | 7 (3, 12) | 1 (-5, 1) |
| **Gut microbiota Score** | | |
| erMEDAS | 10.1 (9.5, 10.6) | 0.2 (-0.4, 0.8) |
| MEDAS | 9.6 (9.1, 9.9) | 0.1 (-0.4, 0.6) |
| DASH | 23.3 (21.5, 25.1) | 0.2 (-0.7, 1.0) |
| HPDI | 54.3 (51.6, 57.3) | 0.0 (-1.3, 1.2) |
| WESTDIET | 35.6 (33.1, 38.2) | -0.1 (-1.3, 1.2) |
| UPDI | 54.9 (52.7, 57.1) | -0.3 (-1.3, 0.8) |
| **Dietary patterns score** | | |
| erMEDAS | 8 (6, 10) | 4 (2, 6) |
| MEDAS | 8 (7, 10) | 3 (1, 4) |
| DASH | 24 (20, 27) | 0 (-4, 4) |
| HPDI | 54 (49, 60) | 0 (-6, 6) |
| WESTDIET | 36 (31, 40) | 0 (-6, 5) |
| UPDI | 55 (50, 60) | -1 (-5, 4) |
| ^1^Median (IQR). **Abbreviations**: BDI-II, Beck Depression Inventory-II; DASH, Dietary Approaches to Stop Hypertension; erMEDAS, Energy-Adjusted Mediterranean Diet Adherence Screener; HPDI, Healthful Plant-Based Diet Index; MEDAS, Mediterranean Diet Adherence Screener; UPDI, Unhealthful Plant-Based Diet Index; WESTDIET, Western Diet Index. | | |

| **Supplementary table S3:** Genera that better predicts adherence to the erMEDAS dietary pattern in the study population according to LMM-LASSO. The table presents the beta coefficients (B) and 95% confidence intervals (95% CI) from linear regression models assessing the association between the erMEDAS score and the relative abundance of each genus. For reference, associations with depressive symptoms measured by the BDI-II score are also shown. Positive beta values indicate a direct association, while negative values reflect an inverse association. | | |
| --- | --- | --- |
| **Genus** | **erMEDAS B (95%CI)** | **BDI-II B (95%CI)** |
| *Barnesiella* | 2.82^x10-4^ (2.15^x10-5^,5.43^x10-4^) | -2.14^x10-5^ (-1.42^x10-4^,9.88^x10-5^) |
| *Eubacterium hallii group* | -3.22^x10-4^ (-7.38^x10-4^,9.47^x10-5^) | 7.03^x10-5^ (-1.19^x10-4^,2.60^x10-4^) |
| *Oscillibacter* | -1.67^x10-5^ (-4.75^x10-5^,1.40^x10-5^) | 2.00^x10-5^ (5.93^x10-6^,3.42^x10-5^) |
| *Unclassified UCG-010* | 1.03^x10-4^ (2.29^x10-5^,1.84^x10-4^) | -4.90^x10-5^ (-8.70^x10-5^,-1.09^x10-5^) |
| *Eubacterium fissicatena group* | -3.84^x10-5^ (-7.97^x10-5^,2.88^x10-6^) | 1.29^x10-5^ (-5.88^x10-6^,3.17^x10-5^) |
| *Dorea* | -2.84^x10-4^ (-5.04^x10-4^,-6.33^x10-5^) | 6.05^x10-5^ (-3.89^x10-5^,1.60^x10-4^) |
| *Lachnospiraceae UCG-010* | 3.79^x10-6^ (-2.72^x10-5^,3.47^x10-5^) | 1.73^x10-6^ (-1.22^x10-5^,1.57^x10-5^) |
| *Eubacterium xylanophilum group* | 5.76^x10-5^ (2.30^x10-5^,9.22^x10-5^) | -4.65^x10-7^ (-1.60^x10-5^,1.50^x10-5^) |
| *Eubacterium eligens group* | 1.30^x10-4^ (-1.66^x10-4^,4.26^x10-4^) | -1.86^x10-5^ (-1.53^x10-4^,1.16^x10-4^) |
| *Lachnospiraceae UCG-003* | 7.19^x10-5^ (1.96^x10-5^,1.24^x10-4^) | -8.66^x10-6^ (-3.32^x10-5^,1.59^x10-5^) |
| *Hungatella* | 2.82^x10-4^ (1.21^x10-4^,4.43^x10-4^) | 7.94^x10-6^ (-6.43^x10-5^,8.02^x10-5^) |
| *Oscillospiraceae NK4A214 group* | 1.71^x10-4^ (1.57^x10-5^,3.25^x10-4^) | 6.19^x10-6^ (-6.41^x10-5^,7.65^x10-5^) |
| *Streptococcus* | -8.83^x10-4^ (-1.45^x10-3^,-3.17^x10-4^) | 1.64^x10-4^ (-1.04^x10-4^,4.32^x10-4^) |
| *Christensenellaceae R-7 group* | 5.50^x10-4^ (9.88^x10-5^,1.00^x10-3^) | -8.50^x10-5^ (-2.95^x10-4^,1.25^x10-4^) |
| *Subdoligranulum* | 3.99^x10-4^ (8.26^x10-6^,7.89^x10-4^) | -8.39^x10-5^ (-2.61^x10-4^,9.31^x10-5^) |
| *Bifidobacterium* | -1.22^x10-3^ (-2.22^x10-3^,-2.09^x10-4^) | 3.76^x10-4^ (-8.61^x10-5^,8.38^x10-4^) |
| *Lachnospiraceae NK4A136 group* | 2.09^x10-4^ (6.83^x10-5^,3.49^x10-4^) | -1.09^x10-5^ (-7.37^x10-5^,5.18^x10-5^) |
| *Butyricicoccus* | 3.40^x10-5^ (-3.81^x10-5^,1.06^x10-4^) | -2.01^x10-5^ (-5.20^x10-5^,1.17^x10-5^) |
| *Flavonifractor* | -4.65^x10-5^ (-8.66^x10-5^,-6.33^x10-6^) | 2.23^x10-6^ (-1.60^x10-5^,2.05^x10-5^) |
| *Eubacterium siraeum group* | 6.24^x10-5^ (-8.18^x10-5^,2.07^x10-4^) | -3.33^x10-5^ (-9.70^x10-5^,3.04^x10-5^) |
| *Eubacterium ruminantium group* | 1.79^x10-4^ (1.50^x10-5^,3.43^x10-4^) | -5.28^x10-7^ (-7.30^x10-5^,7.20^x10-5^) |
| *Lachnospiraceae UCG-001* | 4.80^x10-5^ (1.67^x10-5^,7.94^x10-5^) | 7.10^x10-7^ (-1.34^x10-5^,1.48^x10-5^) |

| **Supplementary table S4:** Genera that better predicts adherence to the MEDAS dietary pattern in the study population according to LMM-LASSO. The table presents the beta coefficients (B) and 95% confidence intervals (95% CI) from linear regression models assessing the association between the MEDAS score and the relative abundance of each genus. For reference, associations with depressive symptoms measured by the BDI-II score are also shown. Positive beta values indicate a direct association, while negative values reflect an inverse association. | | |
| --- | --- | --- |
| **Genus** | **MEDAS B (95%CI)** | **BDI-II B (95%CI)** |
| *Barnesiella* | 4.37^x10-4^ (3.59^x10-5^,8.39^x10-4^) | -2.14^x10-5^ (-1.42^x10-4^,9.88^x10-5^) |
| *Eubacterium hallii group* | -4.06^x10-4^ (-1.04^x10-3^,2.33^x10-4^) | 7.03^x10-5^ (-1.19^x10-4^,2.60^x10-4^) |
| *Clostridium UCG-003* | 4.01^x10-6^ (-5.99^x10-5^,6.79^x10-5^) | 4.49^x10-7^ (-1.85^x10-5^,1.94^x10-5^) |
| *Unclassified UCG-010* | 1.14^x10-4^ (-1.07^x10-5^,2.39^x10-4^) | -4.90^x10-5^ (-8.70^x10-5^,-1.09^x10-5^) |
| *Eubacterium fissicatena group* | -7.69^x10-5^ (-1.40^x10-4^,-1.36^x10-5^) | 1.29^x10-5^ (-5.88^x10-6^,3.17^x10-5^) |
| *Dorea* | -3.81^x10-4^ (-7.18^x10-4^,-4.34^x10-5^) | 6.05^x10-5^ (-3.89^x10-5^,1.60^x10-4^) |
| *Lachnospiraceae UCG-010* | -2.97^x10-5^ (-7.70^x10-5^,1.76^x10-5^) | 1.73^x10-6^ (-1.22^x10-5^,1.57^x10-5^) |
| *Eubacterium xylanophilum group* | 7.99^x10-5^ (2.71^x10-5^,1.33^x10-4^) | -4.65^x10-7^ (-1.60^x10-5^,1.50^x10-5^) |
| *Lachnospiraceae UCG-003* | 5.81^x10-5^ (-2.30^x10-5^,1.39^x10-4^) | -8.66^x10-6^ (-3.32^x10-5^,1.59^x10-5^) |
| *Anaerostipes* | -1.60^x10-3^ (-2.50^x10-3^,-7.02^x10-4^) | 2.94^x10-5^ (-2.38^x10-4^,2.97^x10-4^) |
| *Hungatella* | 3.15^x10-4^ (6.90^x10-5^,5.61^x10-4^) | 7.94^x10-6^ (-6.43^x10-5^,8.02^x10-5^) |
| *Eubacterium coprostanoligenes group* | -6.95^x10-4^ (-1.90^x10-3^,5.15^x10-4^) | 2.23^x10-4^ (-1.27^x10-4^,5.72^x10-4^) |
| *Oscillospiraceae NK4A214 group* | 2.54^x10-4^ (1.68^x10-5^,4.91^x10-4^) | 6.19^x10-6^ (-6.41^x10-5^,7.65^x10-5^) |
| *Streptococcus* | -1.49^x10-3^ (-2.37^x10-3^,-6.15^x10-4^) | 1.64^x10-4^ (-1.04^x10-4^,4.32^x10-4^) |
| *Unclassified Lactobacillaceae* | -2.28^x10-4^ (-4.01^x10-4^,-5.54^x10-5^) | 4.08^x10-5^ (-1.05^x10-5^,9.21^x10-5^) |
| *Subdoligranulum* | 7.25^x10-4^ (1.27^x10-4^,1.32^x10-3^) | -8.39^x10-5^ (-2.61^x10-4^,9.31^x10-5^) |
| *Bifidobacterium* | -1.76^x10-3^ (-3.31^x10-3^,-2.08^x10-4^) | 3.76^x10-4^ (-8.61^x10-5^,8.38^x10-4^) |
| *Slackia* | -3.45^x10-5^ (-9.29^x10-5^,2.39^x10-5^) | 1.21^x10-6^ (-1.63^x10-5^,1.87^x10-5^) |
| *Adlercreutzia* | -2.48^x10-5^ (-7.16^x10-5^,2.19^x10-5^) | 4.81^x10-6^ (-8.88^x10-6^,1.85^x10-5^) |
| *Senegalimassilia* | -6.31^x10-6^ (-1.07^x10-4^,9.45^x10-5^) | -2.20^x10-5^ (-5.23^x10-5^,8.38^x10-6^) |
| *Enterorhabdus* | 5.34^x10-5^ (-6.87^x10-6^,1.14^x10-4^) | 4.88^x10-9^ (-1.77^x10-5^,1.77^x10-5^) |
| *Negativibacillus* | -9.47^x10-5^ (-1.63^x10-4^,-2.63^x10-5^) | -3.83^x10-6^ (-2.40^x10-5^,1.64^x10-5^) |
| *Fusicatenibacter* | -5.71^x10-4^ (-9.42^x10-4^,-1.99^x10-4^) | -2.66^x10-5^ (-1.37^x10-4^,8.35^x10-5^) |
| *Lachnospiraceae NK4A136 group* | 2.66^x10-4^ (5.19^x10-5^,4.81^x10-4^) | -1.09^x10-5^ (-7.37^x10-5^,5.18^x10-5^) |
| *Butyricicoccus* | 9.31^x10-5^ (-1.65^x10-5^,2.03^x10-4^) | -2.01^x10-5^ (-5.20^x10-5^,1.17^x10-5^) |
| *Flavonifractor* | -6.56^x10-5^ (-1.27^x10-4^,-4.04^x10-6^) | 2.23^x10-6^ (-1.60^x10-5^,2.05^x10-5^) |
| *Ruminococcus gauvreauii group* | -7.27^x10-5^ (-1.46^x10-4^,9.07^x10-7^) | 1.66^x10-5^ (-5.44^x10-6^,3.86^x10-5^) |
| *Eubacterium ruminantium group* | 1.73^x10-4^ (-7.70^x10-5^,4.22^x10-4^) | -5.28^x10-7^ (-7.30^x10-5^,7.20^x10-5^) |

| **Supplementary table S5:** Genera that better predicts adherence to the DASH dietary pattern in the study population according to LMM-LASSO. The table presents the beta coefficients (B) and 95% confidence intervals (95% CI) from linear regression models assessing the association between the DASH score and the relative abundance of each genus. For reference, associations with depressive symptoms measured by the BDI-II score are also shown. Positive beta values indicate a direct association, while negative values reflect an inverse association. | | |
| --- | --- | --- |
| **Genus** | **DASH B (95%CI)** | **BDI-II B (95%CI)** |
| *Sporobacter* | 3.24^x10-5^ (-1.35^x10-5^,7.83^x10-5^) | -1.86^x10-5^ (-5.57^x10-5^,1.84^x10-5^) |
| *Lachnospiraceae UCG-010* | -4.18^x10-6^ (-2.13^x10-5^,1.29^x10-5^) | 1.73^x10-6^ (-1.22^x10-5^,1.57^x10-5^) |
| *Eubacterium xylanophilum group* | 3.43^x10-5^ (1.53^x10-5^,5.33^x10-5^) | -4.65^x10-7^ (-1.60^x10-5^,1.50^x10-5^) |
| *Eubacterium eligens group* | 1.80^x10-4^ (1.65^x10-5^,3.44^x10-4^) | -1.86^x10-5^ (-1.53^x10-4^,1.16^x10-4^) |
| *Herbinix* | 3.38^x10-5^ (1.55^x10-5^,5.20^x10-5^) | -6.19^x10-6^ (-2.13^x10-5^,8.93^x10-6^) |
| *Lachnospiraceae UCG-003* | 4.23^x10-5^ (1.29^x10-5^,7.17^x10-5^) | -8.66^x10-6^ (-3.32^x10-5^,1.59^x10-5^) |
| *Hungatella* | 1.60^x10-4^ (7.16^x10-5^,2.48^x10-4^) | 7.94^x10-6^ (-6.43^x10-5^,8.02^x10-5^) |
| *Peptococcus* | 1.57^x10-5^ (3.36^x10-6^,2.80^x10-5^) | -2.61^x10-6^ (-1.29^x10-5^,7.62^x10-6^) |
| *Oscillospiraceae NK4A214 group* | 1.20^x10-4^ (3.48^x10-5^,2.06^x10-4^) | 6.19^x10-6^ (-6.41^x10-5^,7.65^x10-5^) |
| *Sutterella* | -2.45^x10-5^ (-1.11^x10-4^,6.22^x10-5^) | -2.33^x10-5^ (-9.40^x10-5^,4.74^x10-5^) |
| *Erysipelatoclostridium* | -2.26^x10-5^ (-4.32^x10-5^,-2.00^x10-6^) | 2.16^x10-6^ (-1.49^x10-5^,1.93^x10-5^) |
| *Erysipelotrichaceae UCG-003* | -8.94^x10-5^ (-3.27^x10-4^,1.48^x10-4^) | 1.15^x10-4^ (-8.23^x10-5^,3.13^x10-4^) |
| *Unclassified Lactobacillaceae* | -4.67^x10-5^ (-1.09^x10-4^,1.58^x10-5^) | 4.08^x10-5^ (-1.05^x10-5^,9.21^x10-5^) |
| *Christensenellaceae R-7 group* | 1.96^x10-4^ (-5.72^x10-5^,4.50^x10-4^) | -8.50^x10-5^ (-2.95^x10-4^,1.25^x10-4^) |
| *Subdoligranulum* | 2.05^x10-4^ (-1.12^x10-5^,4.21^x10-4^) | -8.39^x10-5^ (-2.61^x10-4^,9.31^x10-5^) |
| *Negativibacillus* | -2.11^x10-5^ (-4.58^x10-5^,3.64^x10-6^) | -3.83^x10-6^ (-2.40^x10-5^,1.64^x10-5^) |
| *Lachnospiraceae FCS020 group* | 8.65^x10-6^ (1.59^x10-6^,1.57^x10-5^) | -4.73^x10-6^ (-1.06^x10-5^,1.10^x10-6^) |
| *Blautia* | -3.59^x10-4^ (-6.78^x10-4^,-4.06^x10-5^) | 1.32^x10-4^ (-1.32^x10-4^,3.95^x10-4^) |
| *Ruminococcus torques group* | -5.89^x10-5^ (-1.18^x10-4^,3.64^x10-7^) | 9.38^x10-6^ (-3.86^x10-5^,5.74^x10-5^) |
| *Lachnospiraceae NK4A136 group* | 1.15^x10-4^ (3.78^x10-5^,1.92^x10-4^) | -1.09^x10-5^ (-7.37^x10-5^,5.18^x10-5^) |
| *Lachnoclostridium* | -4.15^x10-5^ (-8.67^x10-5^,3.66^x10-6^) | 2.67^x10-5^ (-1.05^x10-5^,6.38^x10-5^) |
| *Eubacterium ruminantium group* | 7.29^x10-5^ (-1.67^x10-5^,1.63^x10-4^) | -5.28^x10-7^ (-7.30^x10-5^,7.20^x10-5^) |
| *Lachnospiraceae UCG-001* | 2.18^x10-5^ (4.57^x10-6^,3.91^x10-5^) | 7.10^x10-7^ (-1.34^x10-5^,1.48^x10-5^) |
| *Clostridium UC5.1-2E3* | -2.22^x10-6^ (-1.05^x10-5^,6.04^x10-6^) | 3.19^x10-6^ (-3.62^x10-6^,9.99^x10-6^) |

| **Supplementary table S6:** Genera that better predicts adherence to the HPDI dietary pattern in the study population according to LMM-LASSO. The table presents the beta coefficients (B) and 95% confidence intervals (95% CI) from linear regression models assessing the association between the HPDI score and the relative abundance of each genus. For reference, associations with depressive symptoms measured by the BDI-II score are also shown. Positive beta values indicate a direct association, while negative values reflect an inverse association. | | |
| --- | --- | --- |
| **Genus** | **HPDI B (95%CI)** | **BDI-II B (95%CI)** |
| *Bacteroides* | 7.41^x10-4^ (-1.03^x10-4^,1.58^x10-3^) | -3.93^x10-4^ (-1.51^x10-3^,7.29^x10-4^) |
| *Alistipes* | 2.01^x10-4^ (5.72^x10-5^,3.45^x10-4^) | -8.90^x10-5^ (-2.81^x10-4^,1.03^x10-4^) |
| *Eubacterium fissicatena group* | -1.00^x10-5^ (-2.43^x10-5^,4.18^x10-6^) | 1.29^x10-5^ (-5.88^x10-6^,3.17^x10-5^) |
| *Dorea* | -1.23^x10-4^ (-1.98^x10-4^,-4.78^x10-5^) | 6.05^x10-5^ (-3.89^x10-5^,1.60^x10-4^) |
| *Eubacterium xylanophilum group* | 2.10^x10-5^ (9.26^x10-6^,3.27^x10-5^) | -4.65^x10-7^ (-1.60^x10-5^,1.50^x10-5^) |
| *Eubacterium eligens group* | 1.37^x10-4^ (3.58^x10-5^,2.39^x10-4^) | -1.86^x10-5^ (-1.53^x10-4^,1.16^x10-4^) |
| *Herbinix* | 2.03^x10-5^ (8.92^x10-6^,3.16^x10-5^) | -6.19^x10-6^ (-2.13^x10-5^,8.93^x10-6^) |
| *Anaerostipes* | -2.33^x10-4^ (-4.35^x10-4^,-3.09^x10-5^) | 2.94^x10-5^ (-2.38^x10-4^,2.97^x10-4^) |
| *Hungatella* | 1.26^x10-4^ (7.14^x10-5^,1.80^x10-4^) | 7.94^x10-6^ (-6.43^x10-5^,8.02^x10-5^) |
| *Oscillospiraceae NK4A214 group* | 8.18^x10-5^ (2.88^x10-5^,1.35^x10-4^) | 6.19^x10-6^ (-6.41^x10-5^,7.65^x10-5^) |
| *Erysipelotrichaceae UCG-003* | -1.12^x10-4^ (-2.60^x10-4^,3.65^x10-5^) | 1.15^x10-4^ (-8.23^x10-5^,3.13^x10-4^) |
| *Streptococcus* | -1.87^x10-4^ (-3.87^x10-4^,1.43^x10-5^) | 1.64^x10-4^ (-1.04^x10-4^,4.32^x10-4^) |
| *Bifidobacterium* | -3.38^x10-4^ (-6.86^x10-4^,1.11^x10-5^) | 3.76^x10-4^ (-8.61^x10-5^,8.38^x10-4^) |
| *Adlercreutzia* | -5.79^x10-6^ (-1.62^x10-5^,4.60^x10-6^) | 4.81^x10-6^ (-8.88^x10-6^,1.85^x10-5^) |
| *Blautia* | -2.46^x10-4^ (-4.45^x10-4^,-4.80^x10-5^) | 1.32^x10-4^ (-1.32^x10-4^,3.95^x10-4^) |
| *Lachnospiraceae NK4A136 group* | 1.01^x10-4^ (5.36^x10-5^,1.48^x10-4^) | -1.09^x10-5^ (-7.37^x10-5^,5.18^x10-5^) |

| **Supplementary table S7:** Genera that better predicts adherence to the WESTDIET dietary pattern in the study population according to LMM-LASSO. The table presents the beta coefficients (B) and 95% confidence intervals (95% CI) from linear regression models assessing the association between the WESTDIET score and the relative abundance of each genus. For reference, associations with depressive symptoms measured by the BDI-II score are also shown. Positive beta values indicate a direct association, while negative values reflect an inverse association. | | |
| --- | --- | --- |
| **Genus** | **WESTDIET B (95%CI)** | **BDI-II B (95%CI)** |
| *Alistipes* | -1.66^x10-4^ (-3.23^x10-4^,-8.74^x10-6^) | -8.90^x10-5^ (-2.81^x10-4^,1.03^x10-4^) |
| *Barnesiella* | -8.74^x10-5^ (-1.86^x10-4^,1.16^x10-5^) | -2.14^x10-5^ (-1.42^x10-4^,9.88^x10-5^) |
| *Paraprevotella* | -1.06^x10-4^ (-2.11^x10-4^,-1.92^x10-6^) | 3.18^x10-7^ (-1.27^x10-4^,1.28^x10-4^) |
| *Clostridium UCG-005* | -2.38^x10-5^ (-8.33^x10-5^,3.58^x10-5^) | -3.55^x10-5^ (-1.08^x10-4^,3.69^x10-5^) |
| *Sporobacter* | -4.15^x10-5^ (-7.25^x10-5^,-1.05^x10-5^) | -1.86^x10-5^ (-5.57^x10-5^,1.84^x10-5^) |
| *Clostridium UCG-003* | -1.78^x10-5^ (-3.34^x10-5^,-2.08^x10-6^) | 4.49^x10-7^ (-1.85^x10-5^,1.94^x10-5^) |
| *Unclassified UCG-010* | -2.63^x10-5^ (-5.75^x10-5^,4.78^x10-6^) | -4.90^x10-5^ (-8.70^x10-5^,-1.09^x10-5^) |
| *Eubacterium fissicatena group* | 1.74^x10-5^ (1.82^x10-6^,3.29^x10-5^) | 1.29^x10-5^ (-5.88^x10-6^,3.17^x10-5^) |
| *Dorea* | 1.04^x10-4^ (2.14^x10-5^,1.86^x10-4^) | 6.05^x10-5^ (-3.89^x10-5^,1.60^x10-4^) |
| *Eubacterium xylanophilum group* | -2.63^x10-5^ (-3.92^x10-5^,-1.35^x10-5^) | -4.65^x10-7^ (-1.60^x10-5^,1.50^x10-5^) |
| *Eubacterium eligens group* | -1.62^x10-4^ (-2.73^x10-4^,-5.11^x10-5^) | -1.86^x10-5^ (-1.53^x10-4^,1.16^x10-4^) |
| *Herbinix* | -2.29^x10-5^ (-3.53^x10-5^,-1.05^x10-5^) | -6.19^x10-6^ (-2.13^x10-5^,8.93^x10-6^) |
| *Lachnospiraceae UCG-003* | -2.97^x10-5^ (-4.97^x10-5^,-9.65^x10-6^) | -8.66^x10-6^ (-3.32^x10-5^,1.59^x10-5^) |
| *Hungatella* | -1.50^x10-4^ (-2.09^x10-4^,-9.01^x10-5^) | 7.94^x10-6^ (-6.43^x10-5^,8.02^x10-5^) |
| *Oscillospiraceae NK4A214 group* | -9.72^x10-5^ (-1.55^x10-4^,-3.92^x10-5^) | 6.19^x10-6^ (-6.41^x10-5^,7.65^x10-5^) |
| *Erysipelatoclostridium* | 1.20^x10-5^ (-2.03^x10-6^,2.61^x10-5^) | 2.16^x10-6^ (-1.49^x10-5^,1.93^x10-5^) |
| *Erysipelotrichaceae UCG-003* | 1.33^x10-4^ (-2.92^x10-5^,2.94^x10-4^) | 1.15^x10-4^ (-8.23^x10-5^,3.13^x10-4^) |
| *Streptococcus* | 2.54^x10-4^ (3.58^x10-5^,4.73^x10-4^) | 1.64^x10-4^ (-1.04^x10-4^,4.32^x10-4^) |
| *Unclassified Lactobacillaceae* | 4.67^x10-5^ (4.22^x10-6^,8.91^x10-5^) | 4.08^x10-5^ (-1.05^x10-5^,9.21^x10-5^) |
| *Christensenellaceae R-7 group* | -1.68^x10-4^ (-3.41^x10-4^,4.28^x10-6^) | -8.50^x10-5^ (-2.95^x10-4^,1.25^x10-4^) |
| *Subdoligranulum* | -1.36^x10-4^ (-2.82^x10-4^,1.11^x10-5^) | -8.39^x10-5^ (-2.61^x10-4^,9.31^x10-5^) |
| *Bifidobacterium* | 3.55^x10-4^ (-2.63^x10-5^,7.37^x10-4^) | 3.76^x10-4^ (-8.61^x10-5^,8.38^x10-4^) |
| *Lachnospiraceae FCS020 group* | -5.99^x10-6^ (-1.08^x10-5^,-1.18^x10-6^) | -4.73^x10-6^ (-1.06^x10-5^,1.10^x10-6^) |
| *Blautia* | 2.95^x10-4^ (7.86^x10-5^,5.12^x10-4^) | 1.32^x10-4^ (-1.32^x10-4^,3.95^x10-4^) |
| *Lachnospiraceae NK4A136 group* | -1.08^x10-4^ (-1.60^x10-4^,-5.56^x10-5^) | -1.09^x10-5^ (-7.37^x10-5^,5.18^x10-5^) |
| *Eubacterium ruminantium group* | -6.38^x10-5^ (-1.24^x10-4^,-3.16^x10-6^) | -5.28^x10-7^ (-7.30^x10-5^,7.20^x10-5^) |
| *Lachnospiraceae UCG-001* | -1.57^x10-5^ (-2.74^x10-5^,-4.05^x10-6^) | 7.10^x10-7^ (-1.34^x10-5^,1.48^x10-5^) |

| **Supplementary table 8:** Genera that better predicts adherence to the UPDI dietary pattern in the study population according to LMM-LASSO. The table presents the beta coefficients (B) and 95% confidence intervals (95% CI) from linear regression models assessing the association between the UPDI score and the relative abundance of each genus. For reference, associations with depressive symptoms measured by the BDI-II score are also shown. Positive beta values indicate a direct association, while negative values reflect an inverse association. | | |
| --- | --- | --- |
| **Genus** | **UPDI (95%CI)** | **BDI-II B (95%CI)** |
| *Colidextribacter* | 4.24^x10-6^ (-7.33^x10-6^,1.58^x10-5^) | 2.48^x10-6^ (-9.94^x10-6^,1.49^x10-5^) |
| *Unclassified UCG-010* | -3.40^x10-5^ (-6.87^x10-5^,7.18^x10-7^) | -4.90^x10-5^ (-8.70^x10-5^,-1.09^x10-5^) |
| *Coprococcus* | -4.50^x10-5^ (-1.03^x10-4^,1.28^x10-5^) | -5.77^x10-5^ (-1.20^x10-4^,4.19^x10-6^) |
| *Lachnospiraceae UCG-010* | -1.45^x10-6^ (-1.45^x10-5^,1.15^x10-5^) | 1.73^x10-6^ (-1.22^x10-5^,1.57^x10-5^) |
| *Eubacterium xylanophilum group* | -2.64^x10-5^ (-4.08^x10-5^,-1.20^x10-5^) | -4.65^x10-7^ (-1.60^x10-5^,1.50^x10-5^) |
| *Herbinix* | -2.48^x10-5^ (-3.87^x10-5^,-1.09^x10-5^) | -6.19^x10-6^ (-2.13^x10-5^,8.93^x10-6^) |
| *Lachnospiraceae UCG-003* | -3.01^x10-5^ (-5.25^x10-5^,-7.70^x10-6^) | -8.66^x10-6^ (-3.32^x10-5^,1.59^x10-5^) |
| *Monoglobus* | -1.75^x10-5^ (-3.77^x10-5^,2.62^x10-6^) | 7.68^x10-6^ (-1.40^x10-5^,2.93^x10-5^) |
| *Paeniclostridium* | 3.63^x10-5^ (-2.28^x10-6^,7.48^x10-5^) | -2.80^x10-5^ (-6.94^x10-5^,1.35^x10-5^) |
| *Intestinibacter* | 2.64^x10-5^ (-1.11^x10-5^,6.39^x10-5^) | -2.68^x10-5^ (-6.73^x10-5^,1.37^x10-5^) |
| *Phascolarctobacterium* | 1.60^x10-4^ (9.35^x10-6^,3.10^x10-4^) | 2.35^x10-4^ (7.34^x10-5^,3.96^x10-4^) |
| *Oscillospiraceae NK4A214 group* | -9.67^x10-5^ (-1.62^x10-4^,-3.17^x10-5^) | 6.19^x10-6^ (-6.41^x10-5^,7.65^x10-5^) |
| *Christensenellaceae R-7 group* | -1.21^x10-4^ (-3.14^x10-4^,7.20^x10-5^) | -8.50^x10-5^ (-2.95^x10-4^,1.25^x10-4^) |
| *Subdoligranulum* | -1.04^x10-4^ (-2.68^x10-4^,6.06^x10-5^) | -8.39^x10-5^ (-2.61^x10-4^,9.31^x10-5^) |
| *Negativibacillus* | 1.33^x10-5^ (-5.45^x10-6^,3.22^x10-5^) | -3.83^x10-6^ (-2.40^x10-5^,1.64^x10-5^) |
| *Lachnospiraceae FCS020 group* | -1.17^x10-5^ (-1.71^x10-5^,-6.38^x10-6^) | -4.73^x10-6^ (-1.06^x10-5^,1.10^x10-6^) |
| *Lachnoclostridium* | 7.64^x10-8^ (-3.43^x10-5^,3.45^x10-5^) | 2.67^x10-5^ (-1.05^x10-5^,6.38^x10-5^) |
| *Eubacterium ruminantium group* | -5.80^x10-5^ (-1.26^x10-4^,1.00^x10-5^) | -5.28^x10-7^ (-7.30^x10-5^,7.20^x10-5^) |
| *Lachnospiraceae UCG-001* | -1.76^x10-5^ (-3.07^x10-5^,-4.49^x10-6^) | 7.10^x10-7^ (-1.34^x10-5^,1.48^x10-5^) |
| *Clostridium UC5.1-2E3* | 3.18^x10-6^ (-3.10^x10-6^,9.47^x10-6^) | 3.19^x10-6^ (-3.62^x10-6^,9.99^x10-6^) |

| **Supplementary table 9:** Estimates for the mediation paths (a: Diet → GMS; b: GMS → BDI-II) for both longitudinal associations and between their changes after one year. | | |
| --- | --- | --- |
| **Exposure → Outcome** | **B (95%CI)** | **p-value** |
| *Path A: Dietary pattern score against dietary pattern-GMS* | | |
| erMEDAS → GMS-erMEDAS | 0.067 (0.048. 0.087) | <0.001 |
| MEDAS → GMS-MEDAS | 0.084 (0.064. 0.105) | <0.001 |
| HPDI → GMS-HPDI | 0.431 (0.414. 0.448) | <0.001 |
| UPDI → GMS-UPDI | 0.385 (0.368. 0.402) | <0.001 |
| DASH → GMS-DASH | 0.378 (0.360. 0.396) | <0.001 |
| WESTDIET → GMS-WESTDIET | 0.399 (0.381. 0.416) | <0.001 |
| *Path B: Dietary pattern-GMS against BDI-II* | | |
| GMS-erMEDAS → BDI-II | -0.554 (-0.890. -0.219) | 0.001 |
| GMS-MEDAS → BDI-II | -0.716 (-1.201. -0.231) | 0.004 |
| GMS-HPDI → BDI-II | -0.096 (-0.186. -0.005) | 0.039 |
| GMS-UPDI → BDI-II | 0.067 (-0.052. 0.186) | 0.267 |
| GMS-DASH → BDI-II | -0.216 (-0.370. -0.062) | 0.006 |
| GMS-WESTDIET → BDI-II | 0.161 (0.060. 0.261) | 0.002 |
| *Path A: ΔDietary pattern score against Δdietary pattern-GMS* | | |
| erMEDAS → GMS-erMEDAS | 0.048 (0.017. 0.079) | 0.002 |
| MEDAS → GMS-MEDAS | 0.058 (0.027. 0.090) | <0.001 |
| HPDI → GMS-HPDI | 0.432 (0.405. 0.458) | <0.001 |
| UPDI → GMS-UPDI | 0.385 (0.360. 0.410) | <0.001 |
| DASH → GMS-DASH | 0.381 (0.353. 0.408) | <0.001 |
| WESTDIET → GMS-WESTDIET | 0.382 (0.352. 0.412) | <0.001 |
| *Path B: ΔDietary pattern-GMS against ΔBDI-II* | | |
| GMS-erMEDAS → BDI-II | -0.463 (-0.886. -0.039) | 0.032 |
| GMS-MEDAS → BDI-II | -0.521 (-1.131. 0.088) | 0.093 |
| GMS-HPDI → BDI-II | 0.029 (-0.159. 0.217) | 0.763 |
| GMS-UPDI → BDI-II | -0.145 (-0.368. 0.079) | 0.204 |
| GMS-DASH → BDI-II | 0.090 (-0.181. 0.360) | 0.515 |
| GMS-WESTDIET → BDI-II | 0.018 (-0.156. 0.192) | 0.841 |


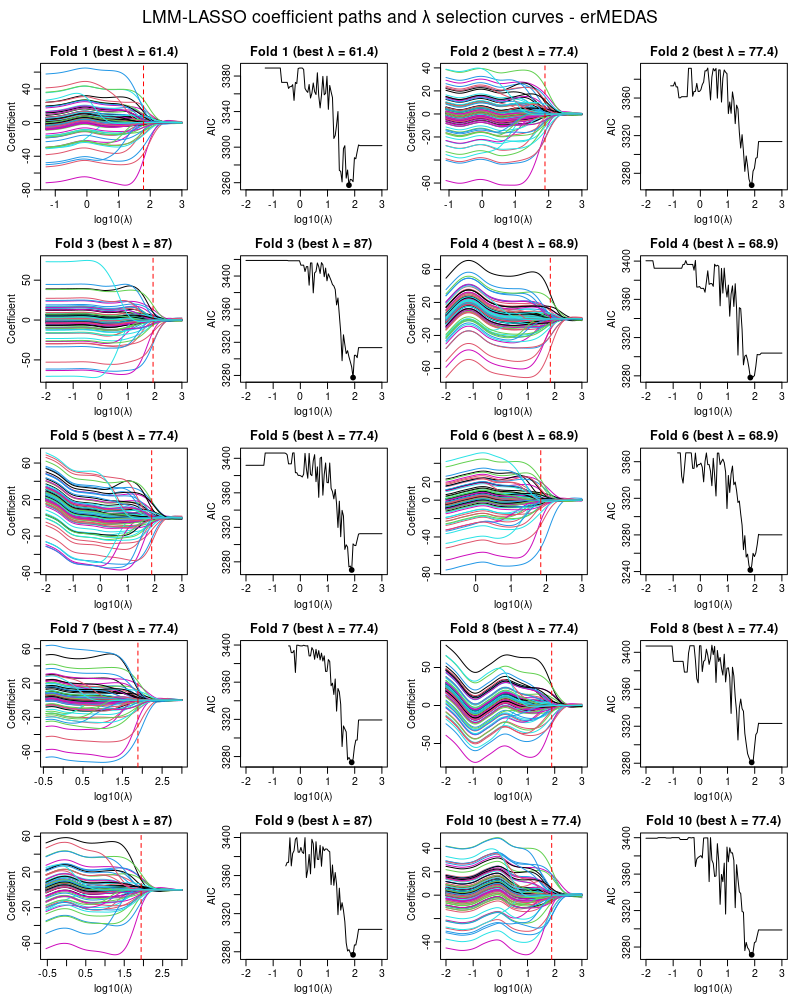
 **Supplementary Figure S1.** LMM-LASSO coefficient paths (left) and λ selection curves (AIC vs log₁₀(λ), right) for the erMEDAS pattern across 10 cross-validation folds. Red vertical lines indicate the AIC-optimal λ in each fold.


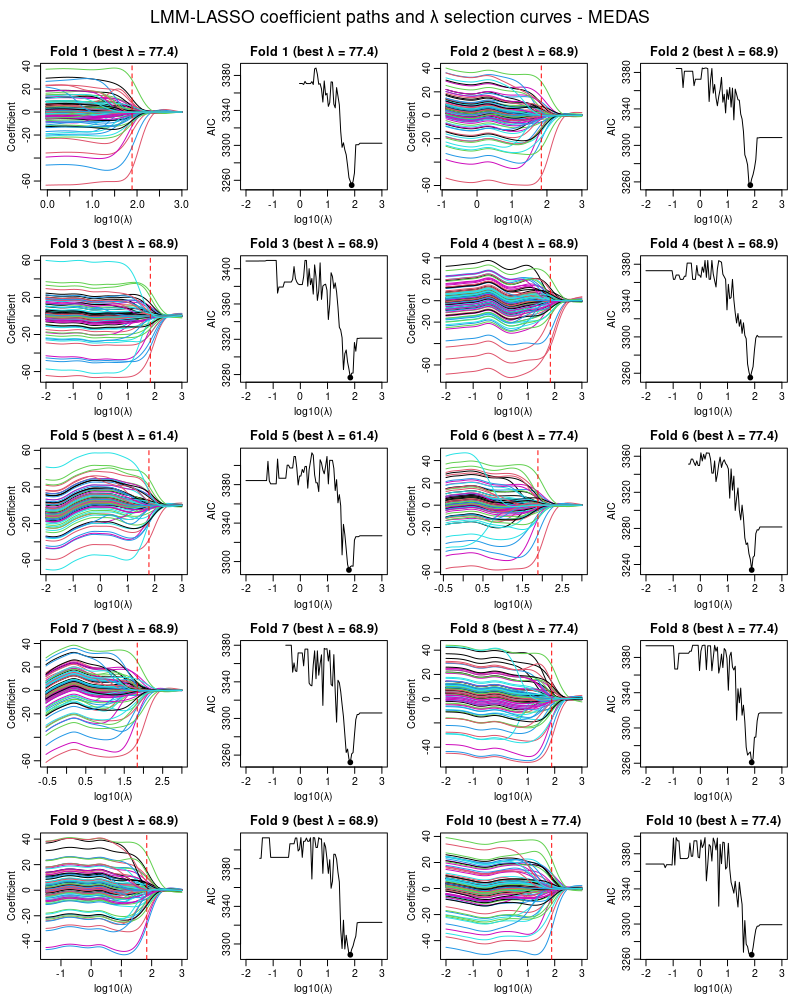
**Supplementary Figure S2.** LMM-LASSO coefficient paths (left) and λ selection curves (AIC vs log₁₀(λ), right) for the MEDAS pattern across 10 cross-validation folds. Red vertical lines indicate the AIC-optimal λ in each fold.


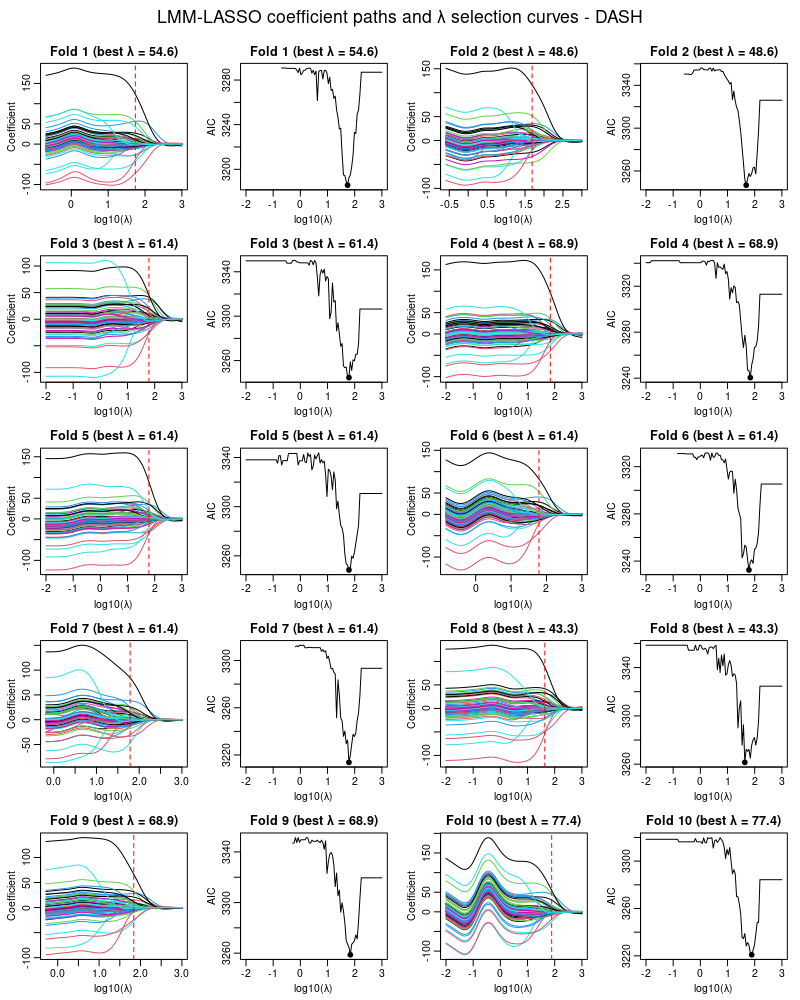
 **Supplementary Figure S3.** LMM-LASSO coefficient paths (left) and λ selection curves (AIC vs log₁₀(λ), right) for the DASH pattern across 10 cross-validation folds. Red vertical lines indicate the AIC-optimal λ in each fold.


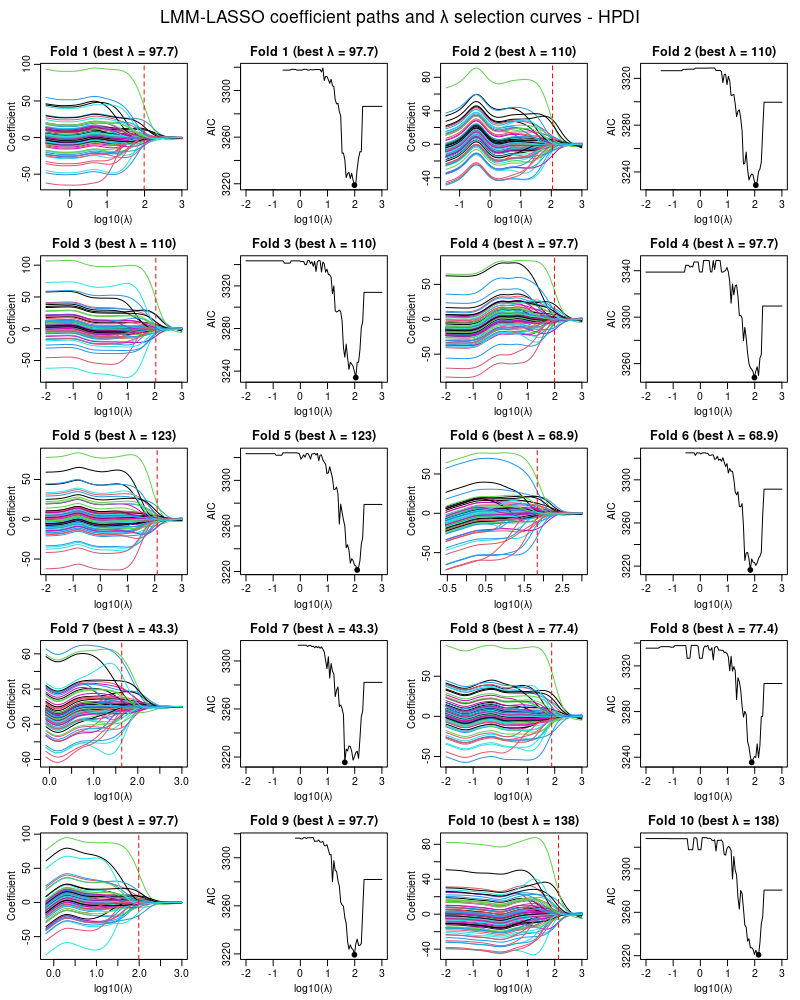
 **Supplementary Figure S4.** LMM-LASSO coefficient paths (left) and λ selection curves (AIC vs log₁₀(λ), right) for the HPDI pattern across 10 cross-validation folds. Red vertical lines indicate the AIC-optimal λ in each fold.
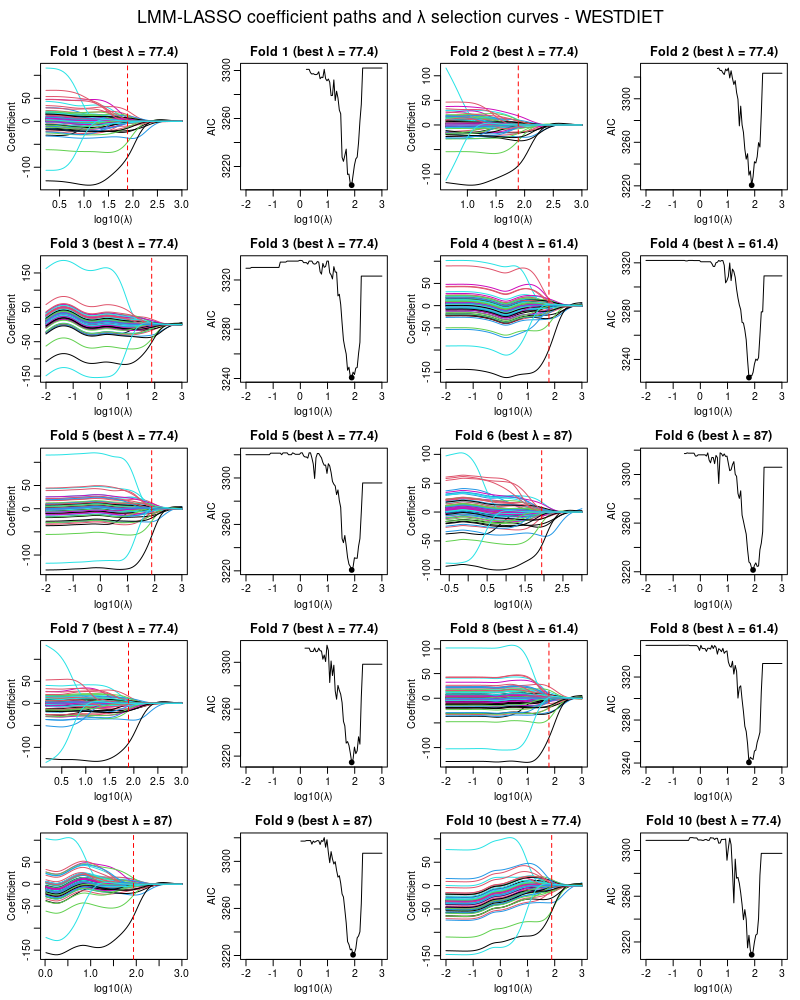
 **Supplementary Figure S5.** LMM-LASSO coefficient paths (left) and λ selection curves (AIC vs log₁₀(λ), right) for the WESTDIET pattern across 10 cross-validation folds. Red vertical lines indicate The AIC-optimal λ in each fold.


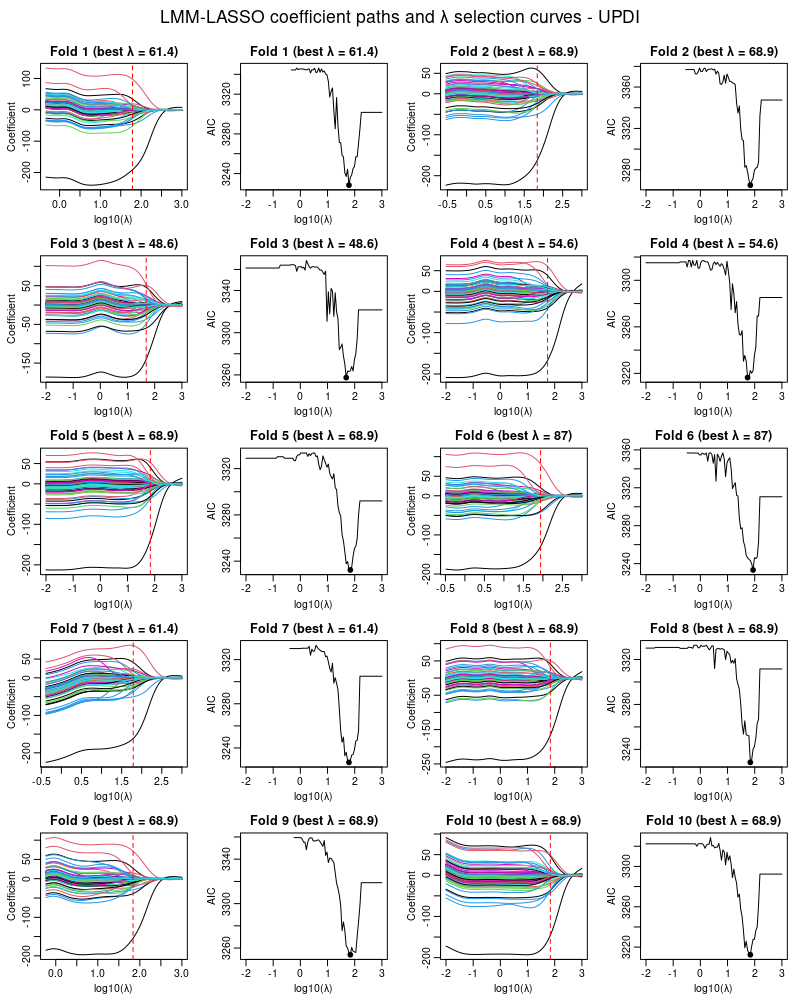
 **Supplementary Figure S6.** LMM-LASSO coefficient paths (left) and λ selection curves (AIC vs log₁₀(λ), right) for the UPDI pattern across 10 cross-validation folds. Red vertical lines indicate the AIC-optimal λ in each fold.
